# Crucial role of dendritic cells in the generation of anti-tumor T-cell responses and immunogenic tumor microenvironment to suppress tumor development

**DOI:** 10.3389/fimmu.2024.1200461

**Published:** 2024-08-14

**Authors:** Moe Tominaga, Tomofumi Uto, Tomohiro Fukaya, Shuya Mitoma, Dieter Riethmacher, Kunihiko Umekita, Yoshihiro Yamashita, Katsuaki Sato

**Affiliations:** ^1^ Division of Immunology, Department of Infectious Diseases, Faculty of Medicine, University of Miyazaki, Miyazaki, Japan; ^2^ Department of Oral and Maxillofacial Surgery, Faculty of Medicine, University of Miyazaki, Miyazaki, Japan; ^3^ Project for Promotion of Cancer Research and Therapeutic Evolution (P-PROMOTE), Japan Agency for Medical Research and Development (AMED), Tokyo, Japan; ^4^ Department of Biomedical Sciences, School of Medicine, Nazarbayev University, Astana, Kazakhstan; ^5^ Division of Respirology, Rheumatology, Infectious Diseases, and Neurology, Department of Internal Medicine, Faculty of Medicine, University of Miyazaki, Miyazaki, Japan; ^6^ Frontier Science Research Center, University of Miyazaki, Miyazaki, Japan

**Keywords:** dendritic cells, tumor development, anti-tumor T-cell responses, tumor microenvironment, myeloid-derived suppressor cells, immunosuppressive mediators

## Abstract

Dendritic cells (DCs) are known as unique professional antigen (Ag)-presenting cells (APCs) to prime naïve T cells for the initiation of adaptive immunity. While DCs are believed to play a pivotal role in generating anti-tumor T-cell responses, the importance of DCs in the protection from the progression of tumors remains elusive. Here, we show how the constitutive deficiency of CD11c^hi^ DCs influences the progression of tumors with the use of binary transgenic mice with constitutive loss of CD11c^hi^ DCs. Constitutive loss of CD11c^hi^ DCs not only enhances the progression of tumors but also reduces the responses of Ag-specific T cells. Furthermore, the congenital deficiency of CD11c^hi^ DCs generates the immunosuppressive tumor microenvironment (TME) that correlates with the marked accumulation of myeloid-derived suppressor cells (MDSCs) and the prominent productions of immunosuppressive mediators. Thus, our findings suggest that CD11c^hi^ DCs are crucial for generating anti-tumor T-cell responses and immunogenic TME to suppress the development of tumors.

## Introduction

Dendritic cells (DCs) are regarded as antigen (Ag)-presenting cells (APCs) that play a pivotal role in linking innate and adaptive immunity (1–3). DCs are divided into functionally distinguishable two subsets, conventional DCs (cDCs) and plasmacytoid DCs (pDCs) (1–3). DCs recognize and process intracellular or extracellular Ags for the presentation of the complexes of the antigenic peptides and major histocompatibility complex (MHC) class I (MHC I) or MHC class II (MHC II), together with costimulatory molecules and cytokines, for differentiation of naïve T cells into effector T (T_eff_) cells (1–3). cDCs are subdivided into type 1 cDC (cDC1) and type 2 cDC (cDC2) with different developmental pathways and functions (1–3). The differentiation of cDC1 depends on interferon (IFN) regulatory factor (IRF) 8 and basic leucine zipper ATF-like transcription factor 3 (Batf3), whereas the development of cDC2 requires IRF4, Notch2, and/or kruppel-like factor (KLF) 4 (1–3). Upon sensing foreign Ags, cDC1 mainly generates type 1 helper T (T_H_1) cells or cytotoxic T lymphocytes (CTLs) through an MHC class II-mediated pathway or MHC I-dependent pathway known as cross-presentation (3). However, cDC2 preferentially induces T_H_2 and T_H_17 cells via the MHC class II-mediated pathway of exogenous Ags (3). Conversely, DCs are suggested to be essential in maintaining immune homeostasis under steady-state and certain environmental conditions by generating immune tolerance through mechanisms involving clonal deletion and anergy of Ag-specific T cells as well as the generation of CD4^+^Foxp3^+^ regulatory T (T_reg_) cells (3).

Accumulating lines of evidence suggest that DCs are crucial for the induction of anti-tumor immunity (4, 5). Tumor-infiltrating DCs are thought to initiate anti-tumor immune responses in tumor-draining lymph nodes (TdLNs) that migrated from tumor tissues after capturing and processing tumor Ags (6–8). A recent analysis of cDC1-deficient mice revealed that cDC1 acts as a distinctive subset capable of cross-presenting tumor Ags to naïve CD8^+^ T cells in TdLNs, resulting in the generation of tumor-specific CTLs for the elimination of tumor (4–6, 8–10). cDC1 is also known as a major producer of interleukin (IL)-12, which elicits T_H_1 responses to participate in the induction of anti-tumor immunity (11). Furthermore, analysis of cDC2-deficient mice showed that cDC2 derived from tumor tissues enhances the ability of cDC1 to induce tumor-specific CTLs through the activation of CD4^+^ T cells in TdLNs (4–6, 12). However, it has been reported that pDCs enhance tumor-specific CTL response generated by cDC1 through the secretion of type I IFN (7, 8). Although the importance of DC subsets in the induction of anti-tumor immunity has been highly appreciated, their role in the progression of tumors remains elusive.

In this study, we examine the impact of the constitutive deficiency of CD11c^hi^ DCs on tumor progression with the use of binary transgenic (Tg) mice that constitutively lacked CD11c^hi^ DCs (13, 14).

## Materials and methods

### Mice

The following 6- to 12-week-old mice were used in this study: C57BL/6 mice (Japan Clea, Tokyo, Japan), B6.Cg-Tg(Itgax-cre)1–1Reiz/J mice (CD11c-Cre mice; The Jackson Laboratory, Bar Harbor, ME, USA) (15), and B6.R26:lacZbpAfloxDTA mice (R-DTA mice) (16). R-DTA mice and CD11c-Cre mice were cross-mated to generate CD11c-Cre:R-DTA mice used as CD11c:DTA mice, and their wild-type (WT) littermates were used as CD11c^hi^ DC-sufficient control mice (14). B6.CD45.1^+^OT-II T-cell receptor (TCR) Tg mice holding ovalbumin (OVA)-specific CD4^+^ T cells (B6.CD45.1^+^OT-II mice) and B6.CD45.1^+^OT-I TCR Tg mice holding OVA-specific CD8^+^ T cells (B6.CD45.1^+^OT-I mice) were generated as described previously (14, 17, 18). All mice were housed under specific pathogen-free conditions in the animal facility, and all experiments were approved by the Animal Experiment Committee and Gene Recombination Experiment Committee at the University of Miyazaki.

### Cell line

OVA-transfected derivative of murine melanoma cell line (B16-OVA) (19) and murine colon adenocarcinoma cell line MC38 (20) were kindly provided by Dr. Shin-ichiro Fujii (RIKEN Center for Integrative Medical Sciences, Japan). B16-OVA and MC38 cells were cultured in RPMI 1640 (Wako, Osaka, Japan) containing an antibiotic-antimycotic (Wako) and 10% heat-inactivated fetal calf serum (FCS; Gibco, Grand Island, NY, USA). The cell line was cultured at 37°C in a humidified atmosphere of 5% CO_2_ and air.

### Cell isolation

Single-cell suspensions were prepared from the spleen (Spl) and inguinal lymph nodes (LNs) used as TdLNs after tumor inoculation as described previously (14, 17, 18, 21, 22). In brief, tissue samples were digested with collagenase type III (Worthington Biochemical, Lakewood, NJ, USA) at 37°C for 20 min and were ground between glass slides. Cell suspensions of Spl were treated with red blood cell lysis buffer (Sigma-Aldrich, St. Louis, MO, USA). Single-cell suspensions of leukocytes were prepared by filtering through a 100-µm cell strainer (BD Biosciences, San Jose, CA, USA). CD4^+^ T cells, CD45.1^+^Vα2^+^OT-II CD4^+^ T cells, or CD45.1^+^Vα2^+^OT-I CD8^+^ T cells were purified from splenocytes of C57BL/6 mice, B6.CD45.1^+^OT-II mice, or B6.CD45.1^+^OT-I mice with mouse CD4 T lymphocyte Enrichment Set-DM (BD Biosciences) or mouse CD8 T lymphocyte Enrichment Set-DM (BD Biosciences). Tumor tissues were ground between glass slides, and CD45^+^ leukocytes were prepared by AutoMACS with mouse CD45 Microbeads (Miltenyi Biotec, Bergisch Gladbach, Germany).

### Flow cytometry

Cells were stained with fluorescein-conjugated monoclonal antibodies (mAbs) after Fc blocking with mAb to CD16/CD32 (clone 2.4G2, BD Biosciences) listed in **Supplementary Table 1** in the **Supplementary Material**. Intracellular staining was performed using the Fixation and Permeabilization kit (eBioscience, San Diego, CA, USA). Analysis of fluorescence staining was performed using FACSVerse flow cytometer (BD Biosciences), CytoFLEX flow cytometer (Beckman Coulter, Brea, CA, USA), and FlowJo software (version 10.9.0; Tree Star, Ashland, OR, USA) according to the gating strategy to identify the leukocytes in LNs, Spl, and tumor tissues as described in **Supplementary Figures 1** **–** **3** in the **Supplementary Material**.

### Quantitative reverse transcription–polymerase chain reaction

Quantitative reverse transcription polymerase chain reaction (RT-qPCR) was performed as described previously (14, 21, 22). Total RNA was extracted from cells by RNeasy plus micro kit (Qiagen, Valencia, CA, USA), and the first-strand cDNA was synthesized with oligo(dT)20 primer using the PrimeScript RT Master Mix (Takara, Shiga, Japan) from total RNA. Transcriptional expression levels were analyzed by using SYBR® Premix Ex Taq II on Thermal Cycler Dice (Takara) with specific primer pairs listed in **Supplementary Table 2** in the **Supplementary Material** after normalization for *Gapdh* expression.

### Treatment with Flt3 inhibitor

Mice were treated daily with or without gilteritinib, an inhibitor of Fms-like tyrosine kinase 3 (Flt3), (SelleckChem, Houston, TX, USA; 200 μg/mouse) (23) by oral gavage using 20-gauge, 1.5-inch feeding needles (Natume, Tokyo, Japan) for 20 days. Spl was obtained from the mice on day 21 after the start of administration.

### Measurement of cytokines

Sera were assayed for IL-1β (R&D Systems, Minneapolis, MN, USA), IL-2 (eBioscience), and IL-15 (R&D Systems) using enzyme-linked immunosorbent assay (ELISA) kits according to the manufacturers’ instructions.

### Tumor growth assay and treatment

WT mice or CD11c:DTA mice were inoculated subcutaneously (s.c.) with B16-OVA (1 × 10^5^) or MC38 (5 × 10^5^) in the right back. Tumor size was measured every day after the inoculation for 8 to 18 days using a digital caliper (CP-15CP; Mitsutoyo, Kanagawa, Japan), and tumor volumes were estimated using the following ellipsoidal formula: length × width × height ×0.52. Spl, TdLNs, and tumor tissues were obtained from mice on day 18 after tumor inoculation. In some experiments, Spl, TdLNs, and tumor tissues were obtained from tumor-bearing WT mice or tumor-bearing CD11c:DTA mice with delayed tumor growth on days 19–21 after tumor inoculation when the values of the tumor volumes of tumor-bearing WT mice as controls were in the range of their mean ± standard deviation (s.d.). For the depletion of Gr-1^+^ cells (24, 25), B16-OVA-bearing mice received anti-Gr-1 mAb (clone RB6–8C5, Bio X Cell, Lebanon, NH, USA; 200 µg/mouse) or control Ab (rat IgG Wako; 200 µg/mouse) by i.p. injections every 3 days for 15 days starting from the day of tumor inoculation until the end of the experiment.

### Immunosuppressive function of myeloid-derived suppressor cells

Gr-1^+^ cells used as myeloid-derived suppressor cells (MDSCs) were collected by biotin-conjugated anti-Gr-1 mAb (clone RB6–8C5, BioLegend) and Streptavidin Particles Plus-DM (BD Biosciences) from Spl of B16-OVA-bearing mice on day 18–21 after tumor inoculation. CD4^+^ T cells obtained from C57BL/6 mice were labeled with eFluor™ 670 (Thermo Fisher Scientific, Waltham, MA, USA; 2.5 µM) for 10 min at 37°C. After washing twice with cold medium, CD4^+^ T cells (2 × 10^4^) were stimulated with or without Dynabeads Mouse T-Activator CD3/CD28 (Thermo Fisher Scientific) in the presence or absence of Gr-1^+^ cells (2 × 10^4^) in a 96-well plate. After 4 days, the gated CD4^+^ T cells were analyzed for eFluor™ 670 dilution to detect the dividing cells by flow cytometry.

### Adoptive transfer

Analysis of Ag-specific priming of CD4^+^ T cells or CD8^+^ T cells *in vivo* was performed as described previously (14, 17, 18). In brief, eFluor™ 670-labeled CD45.1^+^OT-II CD4^+^ T cells or CD45.1^+^OT-I CD8^+^ T cells as described above (each 5 × 10^6^/mouse) were intravenously (i.v.) injected into mice. Subsequently, mice were intraperitoneally (i.p.) injected with OVA protein (A5503, Sigma-Aldrich) at 24 hrs after adoptive transfer. The gated CD45.1^+^OT-II CD4^+^ T cells or CD45.1^+^OT-I CD8^+^ T cells in LNs were analyzed for eFluor™ 670 dilution to detect the dividing cells by flow cytometry at 2 days after immunization. In another experiment, B16-OVA-bearing mice on day 18 after tumor inoculation or untreated naïve mice were i.v. or intratumorally (i.t.) injected with eFluor™ 670-labeled CD45.1^+^OT-II CD4^+^ T cells or CD45.1^+^OT-I CD8^+^ T cells (each 5 × 10^6^/mouse) to detect their accumulation in Spl, TdLNs, or tumor tissues. Analysis of the gated CD45.1^+^OT-II CD4^+^ T cells or CD45.1^+^OT-I CD8^+^ T cells in Spl, TdLNs, and tumor tissues was performed at 2 days after adoptive transfer as described above.

### Statistical analysis

Data are expressed as the mean ± s.d. from three to eight individual samples in a single experiment of at least three independent experiments. Statistical analysis was performed using Prism 9 (GraphPad Software, Boston, MA, USA) using the two-sided unpaired Student’s *t*-test. A P-value for significance was indicated with <0.05 or 0.01.

## Results

### Constitutive deficiency of CD11c^hi^ DCs causes abnormal composition of leukocytes

To clarify the contribution of CD11c^hi^ DCs to the protection from the development of tumors, we generated a mouse model with the constitutive deficiency of CD11c^hi^ DCs by cross-mating CD11c-Cre mice (15) and R-DTA mice harboring the diphtheria toxin (DT) α chain (DTA) under control of a loxP-flanked stop cassette in the ubiquitously expressed ROSA26 locus (16) for producing CD11c-Cre:R-DTA double-Tg mice (referred to as CD11c:DTA mice) (14). CD11c:DTA mice showed almost complete depletion of CD11c^hi^ DCs in LNs and Spl under homeostatic conditions when compared with WT mice (**Supplementary Figures 4**, **5** in the **Supplementary Material**). Similar to the published report (14), the partial reduction of the frequency of pDCs was observed in LNs and Spl of CD11c:DTA mice as compared with WT mice (**Supplementary Figures 4**, **5** in the **Supplementary Material**).

We addressed the influence of the constitutive ablation of CD11c^hi^ DCs on the constituency of leukocytes in lymphoid tissues under steady-state conditions. CD11c:DTA mice showed reduced frequencies of CD4^+^ T cells and CD8^+^ T cells, whereas they exhibited the enhanced proportions of Gr-1^+^CD11b^+^F4/80^−^ polymorphonuclear leukocytes (PMNs) and Gr-1^+^CD11b^+^F4/80^+^ monocytes/macrophages in LNs and Spl when compared with WT mice (**Supplementary Figures 4**, **5** in the **Supplementary Material**). In addition, CD11c:DTA mice showed the enhanced frequency of B220^+^ B cells in LNs (**Supplementary Figure 4** in the **Supplementary Material**).

Taken together, these results indicate that the constitutive deficiency of CD11c^hi^ DCs leads to abnormal composition of leukocytes in lymphoid tissues.

Given that Flt3 ligand (Flt3L) is crucial for the development of various types of leukocytes (26–28), we have previously reported the linking of the enhanced generation of myeloid-lineage cells with massive serum production of Flt3L in CD11c:DTA mice (14). To clarify the role of Flt3L on the enhanced development of Gr-1^+^CD11b^+^F4/80^−^ PMNs and Gr-1^+^CD11b^+^F4/80^+^ monocytes/macrophages under the constitutive absence of CD11c^hi^ DCs, we examined the influence of gilteritinib, an inhibitor of Flt3 (23), on their generation in CD11c:DTA mice. Treatment of CD11c:DTA mice with gilteritinib inhibited the enhanced generation of Gr-1^+^CD11b^+^F4/80^−^ PMNs and Gr-1^+^CD11b^+^F4/80^+^ monocytes/macrophages in LNs and Spl under homeostatic conditions (**Supplementary Figure 6** in the **Supplementary Material**).

Collectively, these results indicate that Flt3L is required for the enhanced generation of PMNs and monocytes/macrophages under the constitutive loss of CD11c^hi^ DCs.

We also examined the expression level of CD11c on leukocytes in LNs and Spl. We showed an expression of CD11c on I-A/I-E^hi^CD11c^+^XCR1^+^SIRPα^−^ migratory cDC1, I-A/I-E^hi^CD11c^+^XCR1^−^SIRPα^+^ migratory cDC2, I-A/I-E^+^CD11c^+^XCR1^+^SIRPα^−^ resident cDC1, I-A/I-E^+^CD11c^+^XCR1^−^SIRPα^+^ resident cDC2, I-A/I-E^hi^CD11c^+^CD207^+^XCR1^−^SIRPα^+^ Langerhans cells (LCs) (29), and I-A/I-E^hi^CD11c^+^XCR1^−^SIRPα^+^CD64^+^ monocyte-derived DCs (Mo-DCs)/DC3 (30) in LNs as well as I-A/I-E^+^CD11c^+^XCR1^+^SIRPα^−^ cDC1 and I-A/I-E^+^CD11c^+^XCR1^−^SIRPα^+^ cDC2 in Spl (**Supplementary Figure 7** in the **Supplementary Material**). In addition, CD11c^+^B220^+^Siglec-H^+^NK1.1^−^ pDCs showed a higher expression of CD11c than CD11c^+^B220^+^Siglec-H^−^NK1.1^+^ natural killer (NK) cells (31, 32) in LNs and Spl, while their expression levels were lower than those of cDC subsets (**Supplementary Figure 7** in the **Supplementary Material**). We did not observe an expression of CD11c on CD3^−^CD19^−^NK1.1^+^NKp46^+^CD49a^−^CD49b^+^ NK cells (30), CD3^−^CD19^−^NK1.1^+^NKp46^+^CD49a^+^CD49b^−^ group 1 innate lymphoid cells (ILC1s) (33), and CD3^−^CD19^−^CD127^+^RORγt^+^ ILC3s (34) in LNs and Spl in WT mice (**Supplementary Figure 7** in the **Supplementary Material**).

We further examined the constituencies of the subsets of leukocytes in LNs and Spl in CD11c:DTA mice. CD11c:DTA mice showed the marked reduction of I-A/I-E^hi^CD11c^+^XCR1^+^SIRPα^−^ migratory cDC1, I-A/I-E^hi^CD11c^+^XCR1^−^SIRPα^+^ migratory cDC2, I-A/I-E^+^CD11c^+^XCR1^+^SIRPα^−^ resident cDC1, I-A/I-E^+^CD11c^+^XCR1^−^SIRPα^+^ resident cDC2, I-A/I-E^hi^CD11c^+^CD207^+^XCR1^−^SIRPα^+^ LCs, and I-A/I-E^hi^CD11c^+^XCR1^−^SIRPα^+^CD64^+^ Mo-DCs/DC3 in LNs as well as I-A/I-E^+^CD11c^+^XCR1^+^SIRPα^−^ cDC1 and I-A/I-E^+^CD11c^+^XCR1^−^SIRPα^+^ cDC2 in Spl (**Supplementary Figures 8A–L**, **9A–C** in the **Supplementary Material**). In addition, CD11c:DTA mice exhibited a milder reduction of CD11c^+^B220^+^Siglec-H^+^NK1.1^−^ pDCs than cDC subsets in LNs, whereas their similar significant reductions were observed in Spl (**Supplementary Figures 8M–O**, **9D–F** in the **Supplementary Material**). In contrast, CD11c:DTA mice had normal constituencies of CD11c^+^B220^+^Siglec-H^−^NK1.1^+^ NK cells (35), CD3^−^CD19^−^NK1.1^+^NKp46^+^CD49a^−^CD49b^+^ NK cells, and CD3^−^CD19^−^NK1.1^+^NKp46^+^CD49a^+^CD49b^−^ ILC1s in LNs and Spl (**Supplementary Figures 8M–R**, **9D–I** in the **Supplementary Material**). CD11c:DTA mice also showed a reduction of CD3^−^CD19^−^CD127^+^RORγt^+^ ILC3s in LNs, but not Spl, as compared with WT mice (**Supplementary Figures 8S–U**, **9J–L** in the **Supplementary Material**). We also observed a higher production of IL-2 or lower productions of IL-1β and IL-15 in CD11c:DTA mice than in WT mice (**Supplementary Figure 9M** in the **Supplementary Material**).

Taken together, these results indicate that CD11c^hi^ DC subsets are mainly ablated in lymphoid tissues in CD11c:DTA mice.

### Congenital deficiency of CD11c^hi^ DCs enhances tumor development and accumulation of MDSCs in tumor tissues

**Figure 1:**
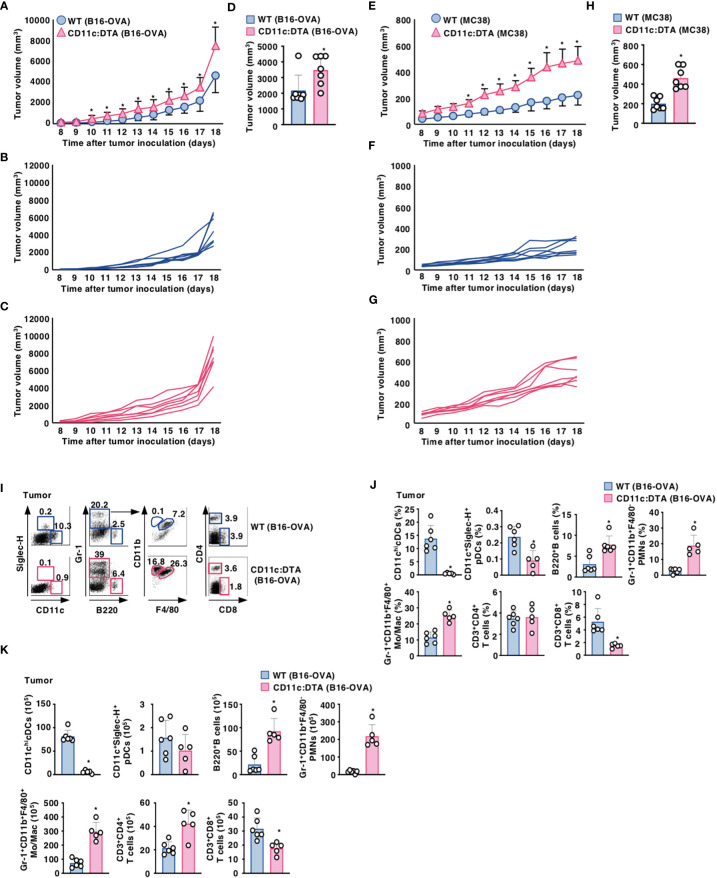
Deficiency of CD11c^hi^ DCs promotes the progression of tumors. **(A–D)** WT mice and CD11c:DTA mice were inoculated with B16-OVA, and tumor growth was monitored. **(A–C)** Average tumor volume curves of WT mice and CD11c:DTA mice **(A)** or tumor volume curves of individual WT mice **(B)** and CD11c:DTA mice **(C)** for 18 days. **(D)** Tumor volume on 17 days; n = 7 per group. *P < 0.05 compared with WT mice by two-sided unpaired Student’s *t*-test. **(E–H)** WT mice and CD11c:DTA mice were inoculated with MC38, and tumor growth was monitored. **(E–G)** Average tumor volume curves of WT mice and CD11c:DTA mice **(E)** or tumor volume curves of individual WT mice **(F)** and CD11c:DTA mice **(G)** for 18 days. **(H)** Tumor volume on 17 days; n = 7 per group. *P < 0.05 compared with WT mice by two-sided unpaired Student’s *t*-test. **(I**–**K)** WT mice and CD11c:DTA mice were inoculated with B16-OVA. Cell surface expression profile **(I)**, proportion **(J)**, and absolute number **(K)** of leukocytes in tumor tissues on days 18–21 after tumor inoculation. Data are obtained from five to six individual samples in a single experiment. Numbers in the dot plot represent the proportion of the indicated cell populations among leukocytes. *P < 0.05 compared with WT mice by two-sided unpaired Student’s *t*-test. All data are representative of at least three independent experiments.

We utilized the experimental model of a murine melanoma tumor cell line with poor immunogenicity that was engineered to express OVA (B16-OVA) (19). When compared with WT mice, CD11c:DTA mice displayed a significant progression of tumor growth for the duration of the experiment (**Figures 1A–D**). We also observed that CD11c:DTA mice exhibited an enhanced development of murine colon adenocarcinoma cell line MC38 known as immunogenic tumor (20) compared with WT mice (**Figures 1E–H**). Therefore, these results indicate that the congenital deficiency of CD11c^hi^ DCs causes the enhanced progression of tumors.

We also compared the cellularity of tumor-infiltrating leukocytes between WT mice and CD11c:DTA mice under tumor-bearing conditions. When compared with tumor-bearing WT mice, tumor-bearing CD11c:DTA mice exhibited the marked infiltrations of Gr-1^+^CD11b^+^F4/80^−^ PMNs, Gr-1^+^CD11b^+^F4/80^+^ monocytes/macrophages, and B220^+^ B cells, whereas they displayed the significant reduction in the accumulations of CD11c^hi^ cDCs, CD11c^+^Siglec-H^+^ pDCs, and CD3^+^CD8^+^ T cells (**Figures 1I–K**). Similar differences in the cellular compositions in TdLN and Spl between WT mice and CD11c:DTA mice were observed under tumor-bearing conditions when compared with those under steady-state conditions (**Supplementary Figures 10**, **11** in the **Supplementary Material**).

Furthermore, tumor-bearing CD11c:DTA mice exhibited a significant reduction of I-A/I-E^hi^CD11c^+^XCR1^+^SIRPα^−^ migratory cDC1 and I-A/I-E^hi^CD11c^+^XCR1^−^SIRPα^+^ migratory cDC2, I-A/I-E^hi^CD11c^+^CD207^+^XCR1^−^SIRPα^+^ LCs, and I-A/I-E^hi^CD11c^+^XCR1^−^SIRPα^+^CD64^+^ Mo-DCs/DC3 in tumor tissues, I-A/I-E^hi^CD11c^+^XCR1^+^SIRPα^−^ migratory cDC1 and I-A/I-E^hi^CD11c^+^XCR1^−^SIRPα^+^ migratory cDC2, I-A/I-E^+^CD11c^+^XCR1^+^SIRPα^−^ resident cDC1, I-A/I-E^+^CD11c^+^XCR1^−^SIRPα^+^ resident cDC2, I-A/I-E^hi^CD11c^+^CD207^+^XCR1^−^SIRPα^+^ LCs, and I-A/I-E^hi^CD11c^+^XCR1^−^SIRPα^+^CD64^+^ Mo-DCs/DC3 in TdLNs as well as I-A/I-E^+^CD11c^+^XCR1^+^SIRPα^−^ cDC1 and I-A/I-E^+^CD11c^+^XCR1^−^SIRPα^+^ cDC2 in Spl compared with those in tumor-bearing WT mice (**Supplementary Figures 12**, **13A–O** in the **Supplementary Material**). Tumor-bearing CD11c:DTA mice also showed a reduction of CD3^−^CD19^−^CD127^+^RORγt^+^ ILC3s in TdLNs as compared with tumor-bearing WT mice (**Supplementary Figures 13P–R** in the **Supplementary Material**). However, tumor-bearing CD11c:DTA mice exhibited higher accumulation of CD11b^+^F4/80^+^CD80^+^ M1-like macrophages (36–38) in TdLNs as well as CD11b^+^F4/80^+^CD80^+^ M1-like macrophages and CD11b^+^F4/80^+^CD206^+^ M2-like macrophages (36–38) in Spl than tumor-bearing WT mice (**Supplementary Figure 14** in the **Supplementary Material**). We also showed that Gr-1^+^ cells composed of Gr-1^+^CD11b^+^F4/80^−^ PMNs and Gr-1^+^CD11b^+^F4/80^+^ monocytes/macrophages in Spl in tumor-bearing WT mice and tumor-bearing CD11c:DTA mice had a similar inhibitory effect on the division of anti-CD3/CD28-stimulated eFluor™ 670-labeled CD4^+^ T cells (**Supplementary Figures 15A, B** in the **Supplementary Material**), indicating that they act as MDSCs (39).

Taken together, these results indicate that the constitutive loss of CD11c^hi^ DCs enhances the accumulation of MDSCs in tumor tissues under tumor-bearing conditions.

It has been shown that MDSCs exhibited an immunosuppressive potential to support tumor progression (40–42). To clarify the contribution of MDSCs to the development of tumors, we addressed the effect of the depletion of MDSCs by anti-Gr-1 mAb (24, 25) on the progression of tumor growth in CD11c:DTA mice. The elimination of MDSCs by anti-Gr-1 mAb exhibited a more dramatic reduction of tumor growth in CD11c:DTA mice than tumor-bearing WT mice (**Supplementary Figures 15C–F** in the **Supplementary Material**), probably due to a more prominent accumulation of MDSCs in tumor tissues (**Figures 1I–K**).

Taken together, these results indicate that MDSCs are involved in the enhanced development of tumors under the constitutive loss of CD11c^hi^ DCs.

### Constitutive loss of CD11c^hi^ DCs affects the activation status of T cells

**Figure 2:**
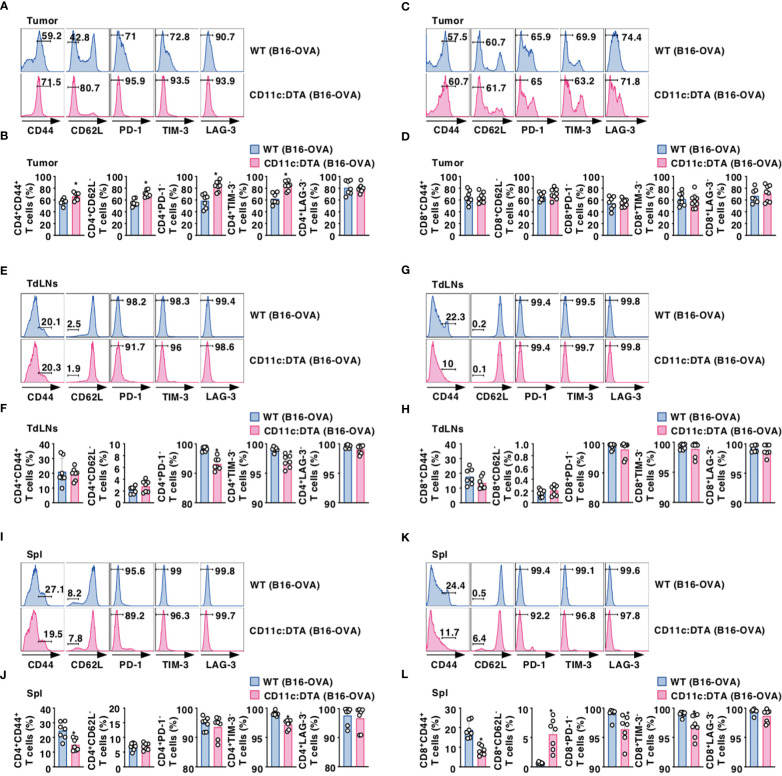
Deficiency of CD11c^hi^ DCs affects the activation status of T cells under tumor-bearing conditions. WT mice and CD11c:DTA mice were inoculated with B16-OVA. Cell surface expression profile **(A, C, E, G, I, K)** and proportion **(B, D, F, H, J, L)** of CD4^+^ T cells **(A, B, E, F, I, J)** and CD8^+^ T cells **(C, D, G, H, K, L)** in tumor tissues **(A–D)**, TdLNs **(E–H)**, and Spl **(I–L)** on days 18–21 after tumor inoculation. Data are obtained from five to eight individual samples in a single experiment. Numbers in the histogram represent the proportion of the indicated cell populations. *P < 0.05 compared with WT mice by two-sided unpaired Student’s *t*-test. All data are representative of at least three independent experiments.

We compared the activation status of T cells in lymphoid tissues between WT mice and CD11c:DTA mice. CD11c:DTA mice had reduced fractions of CD4^+^ T cells and CD8^+^ T cells expressing CD44 as a marker for T-cell activation (43) in LNs and Spl as compared with WT mice in the homeostatic conditions (**Supplementary Figures 16**, **17** in the **Supplementary Material**). Furthermore, CD4^+^ T cells and CD8^+^ T cells in LNs and Spl did not express any immune checkpoint molecules including programmed death (PD)-1, T-cell immunoglobulin and mucin-domain containing-3 (TIM-3), and lymphocyte activation gene (LAG)-3 in both WT mice and CD11c:DTA mice under steady-state conditions (**Supplementary Figures 16**, **17** in the **Supplementary Material**). However, tumor-infiltrating CD4^+^ tumor-infiltrating lymphocytes (TILs) and CD8^+^ TILs obtained from tumor-bearing WT mice not only had the marked proportions of CD4^+^CD44^+^CD62L^−^ T cell subset and CD8^+^CD44^+^CD62L^−^ T cell subset, which function as effector memory T (T_EM_) cells (44), but also expressed PD-1, TIM-3, and LAG-3 (**Figures 2A–D**; **Supplementary Figures 18A, B** in the **Supplementary Material**). In contrast, CD11c:DTA mice had a higher proportion of CD4^+^CD44^+^CD62L^−^ T cells in tumor tissues than WT mice (**Figures 2A, B**; **Supplementary Figure 18A** in the **Supplementary Material**). Interestingly, CD4^+^ TILs, but not CD8^+^ TILs, obtained from CD11c:DTA mice showed lower expressions of PD-1 and TIM-3 than those obtained from WT mice (**Figures 2A–D**; **Supplementary Figures 18A, B** in the **Supplementary Material**).

CD4^+^ T cells and CD8^+^ T cells displayed an enhanced expression of CD44 in TdLNs and Spl in WT mice during tumor progression (**Figures 2E–L**; **Supplementary Figure 18C–F** in the **Supplementary Material**). When compared with tumor-bearing WT mice, CD4^+^ T cells, but not CD8^+^ T cells, obtained from tumor-bearing CD11c:DTA mice exhibited enhanced expressions of PD-1 and TIM-3 in TdLNs (**Figures 2E–H**; **Supplementary Figures 18C, D** in the **Supplementary Material**). Similar to the homeostatic conditions, CD11c:DTA mice showed reduced or enhanced fractions of CD4^+^CD44^+^ T cells and CD8^+^CD44^+^ T cells or CD8^+^CD62L^−^ T cells in Spl as compared with WT mice under tumor-bearing conditions (**Figures 2I–L**; **Supplementary Figures 18E, F** in the **Supplementary Material**). Furthermore, tumor-bearing CD11c:DTA mice showed an enhanced expression of TIM-3 on CD4^+^ T cells and PD-1 and TIM-3 on CD8^+^ T cells in Spl as compared with those obtained from tumor-bearing WT mice (**Figures 2I–L**; **Supplementary Figures 18E, F** in the **Supplementary Material**). Collectively, these results indicate that the constitutive loss of CD11c^hi^ DCs alters the activation status of T cells under tumor-bearing conditions.

### Constitutive absence of CD11c^hi^ DCs affects Ag-specific responses of T cells

To clarify the role of CD11c^hi^ DCs in the Ag-specific T-cell priming, WT mice and CD11c:DTA mice were adoptively transferred with eFluor™ 670-labeled OT-II CD4^+^ T cells or OT-I CD8^+^ T cells expressing the OVA-specific TCR (14, 17, 18) before systemic injection of OVA protein to analyze their Ag-specific division in LNs. In contrast to the prominent division of OT-II CD4^+^ T cells or OT-I CD8^+^ T cells in LNs in WT mice after systemic administration of OVA protein, their responses were significantly reduced in CD11c:DTA mice (**Supplementary Figure 19** in the **Supplementary Material**).

**Figure 3:**
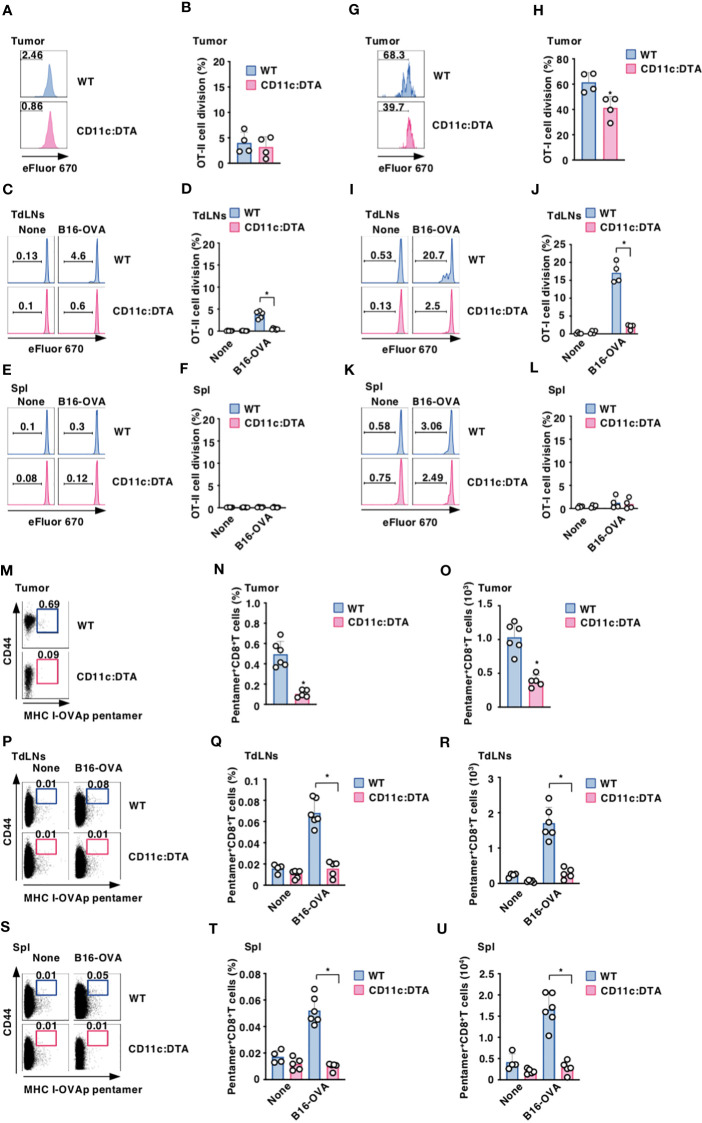
Deficiency of CD11c^hi^ DCs decreases the generation of Ag-specific T_eff_ cells under tumor-bearing conditions. **(A–L)** WT mice and CD11c:DTA mice that had been inoculated with or without B16-OVA were adoptively transferred i.t. **(A, B, G, H)** or i.v. **(C–F, I–L)** with eFluor™ 670-labeled CD45.1^+^OT-II CD4^+^ T cells **(A–F)** or CD45.1^+^OT-I CD8^+^ T cells **(G–L)**. Cell dividing profile **(A, C, E, G, I, K)** and proportion **(B, D, F, H, J, L)** in tumor tissues **(A, B, G, H)**, TdLNs **(C, D, I, J)**, and Spl **(E, F, K, L)** at 2 days after the administration. Data are obtained from four to six individual samples in a single experiment. Numbers in the histogram represent the proportion of the dividing cells. *P < 0.05 compared with WT mice by two-sided unpaired Student’s *t*-test. **(M–U)** WT mice and CD11c:DTA mice were inoculated with or without B16-OVA. Cell surface expression profile **(M, P, S),** proportion **(N, Q, T)**, and absolute number **(O, R, U)** of MHC I-OVA pentamer^+^CD44^high^CD8^+^ T cells among CD8^+^ T cells in tumor tissues **(M–O)**, TdLNs **(P–R)**, and Spl **(S–U)** on days 18–21 after tumor inoculation. Data are obtained from four to six individual samples in a single experiment. Numbers in the dot plot represent the proportion of the indicated cell populations. *P < 0.05 compared with WT mice by two-sided unpaired Student’s *t*-test. All data are representative of at least three independent experiments.

To determine the influence of the constitutive elimination of CD11c^hi^ DCs in the initiation of Ag-specific T-cell responses in tumor-bearing mice, eFluor™ 670-labeled OT-II CD4^+^ T cells or OT-I CD8^+^ T cells were adaptively i.t. or i.v. transferred into B16-OVA-bearing mice to determine their Ag-specific division in tumor tissues or TdLNs and Spl, respectively. While tumor-bearing WT mice exhibited Ag-specific division of OT-I CD8^+^ T cells in tumor tissues and TdLNs as well as OT-II CD4^+^ T cells in TdLNs, they did not show the apparent responses of OT-II CD4^+^ T cells in tumor tissues and Spl as well as OT-I CD8^+^ T cells in Spl (**Figures 3A–L**). However, tumor-bearing CD11c:DTA mice displayed the profound reduction of Ag-specific divisions of OT-I CD8^+^ T cells in tumor tissues and TdLNs as well as OT-II CD4^+^ T cells in TdLNs as compared with tumor-bearing WT mice (**Figures 3C, D, G–J**).

To examine the effect of the constitutive elimination of CD11c^hi^ DCs on the generation of Ag-specific CTLs, we evaluated the occurrence of Ag-specific CD8^+^ T cells based on binding with the MHC I-OVA pentamer in tumor-bearing mice. MHC I-OVA pentamer^+^CD44^high^CD8^+^ T cells were detected in tumor tissues, TdLNs, and Spl in tumor-bearing WT mice (**Figures 3M–U**). However, tumor-bearing CD11c:DTA mice showed a marked reduction of MHC I-OVA pentamer^+^CD44^high^CD8^+^ T cells in tumor tissues, TdLNs, and Spl as compared with tumor-bearing WT mice (**Figures 3M–U**). Similarly, tumor-bearing CD11c:DTA mice exhibited a lower frequency of MHC I-gp100 tetramer^+^CD44^high^CD8^+^ T cells in tumor tissues than tumor-bearing WT mice (**Supplementary Figure 20** in the **Supplementary Material**). Taken together, these results indicate that the congenital deficiency of CD11c^hi^ DCs diminishes Ag-specific responses of T cells under tumor-bearing conditions.

### Constitutive absence of CD11c^hi^ DCs affects the appearance of CD4^+^Foxp3^+^ T_reg_ cells

**Figure 4:**
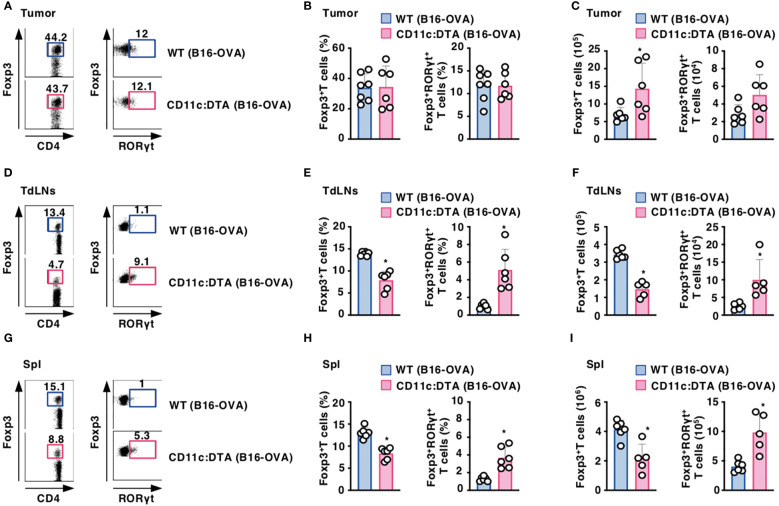
Deficiency of CD11c^hi^ DCs affects the proportion of CD4^+^Foxp3^+^ T_reg_ cells under tumor-bearing conditions. WT mice and CD11c:DTA mice were inoculated with B16-OVA. Cell surface expression profile **(A, D, G)**, proportion **(B, E, H)**, and absolute number **(C, F, I)** of CD4^+^Foxp3^+^ T cells and CD4^+^Foxp3^+^RORγt^+^ T cells among CD4^+^ T cells in tumor tissues **(A–C)**, TdLNs **(D–F)**, and Spl **(G–I)** on days 18–21 after tumor inoculation. Data are obtained from five to seven individual samples in a single experiment. Numbers in the dot plot represent the proportion of the indicated cell populations. *P < 0.05 compared with WT mice by two-sided unpaired Student’s *t*-test. All data are representative of at least three independent experiments.

To address the effect of the constitutive absence of CD11c^hi^ DCs on the composition of CD4^+^Foxp3^+^ T_reg_ cells, we compared their proportion between WT mice and CD11c:DTA mice. CD11c:DTA mice displayed a reduced frequency of CD4^+^Foxp3^+^ T_reg_ cells, whereas they exhibited the enhanced frequency of CD4^+^Foxp3^+^ retinoic acid-related orphan receptor γt (RORγt)^+^ T_reg_ cells (45, 46), in LNs and Spl when compared with WT mice in homeostatic conditions (**Supplementary Figure 21** in the **Supplementary Material**). However, tumor-bearing WT mice and CD11c:DTA mice showed comparable accumulations of CD4^+^Foxp3^+^ T_reg_ cells and CD4^+^Foxp3^+^RORγt^+^ T_reg_ cells in tumor tissues (**Figures 4A–C**). Similar to the homeostatic conditions, tumor-bearing CD11c:DTA mice showed a reduced proportion of CD4^+^Foxp3^+^ T_reg_ cells and an enhanced proportion of CD4^+^Foxp3^+^RORγt^+^ T_reg_ cells in TdLNs and Spl as compared with tumor-bearing WT mice (**Figures 4D–I**). Taken together, these results suggest that the constitutive elimination of CD11c^hi^ DCs alters the composition of CD4^+^Foxp3^+^ T_reg_ cells in lymphoid tissues under homeostatic and tumor-bearing conditions.

### Constitutive loss of CD11c^hi^ DCs enhances the production of immunosuppressive mediators in tumor tissues

**Figure 5:**
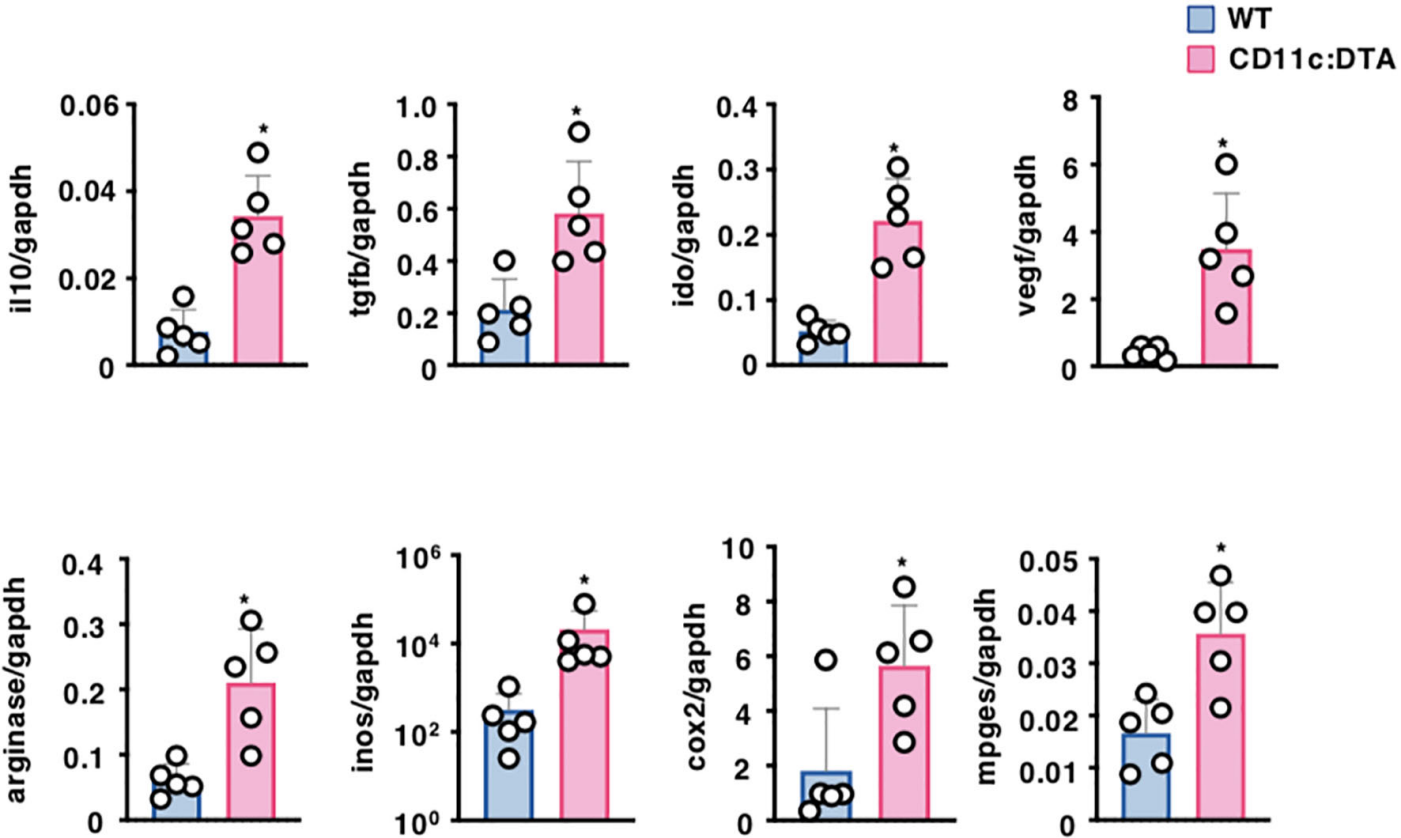
Deficiency of CD11c^hi^ DCs affects the formation of the TME. WT mice and CD11c:DTA mice were inoculated with B16-OVA. Transcriptional expressions of the immunosuppressive molecules in tumor tissues on days 18–21 after tumor inoculation. Data are obtained from five individual samples in a single experiment. *P < 0.05 compared with WT mice by two-sided unpaired Student’s *t*-test. All data are representative of at least three independent experiments.

Tumor cells and tumor-infiltrating immune cells secrete several soluble factors that are thought to generate the immunosuppressive tumor microenvironment (TME), which attenuates the anti-tumor immune responses, leading to progressive tumor growth (47–49). We therefore compared the transcriptional expressions of the immunosuppressive cytokines and metabolite-generating enzymes in tumor tissues between tumor-bearing WT mice and CD11c:DTA mice (**Figure 5**). Tumor tissues obtained from tumor-bearing CD11c:DTA mice exhibited higher transcriptional expressions of IL-10, transforming growth factor (TGF)-β, indoleamine 2,3-dioxygenase (IDO), vascular endothelial growth factor (VEGF), arginase, inducible nitric oxide synthase (iNOS), cyclooxygenase (COX)-2, and membrane-associated prostaglandin E2 synthase (mPGES) than tumor-bearing WT mice. Collectively, these results indicate that the congenital deficiency of CD11c^hi^ DCs promotes the production of immunosuppressive mediators in tumor tissues.

## Discussion

While DCs are reportedly critical for the control of anti-tumor immunity, the contribution of DCs to the protection from the development of tumors remains elusive. In the present study, we demonstrated that the congenital deficiency of CD11c^hi^ DCs promoted the progression of tumors associated with the impaired responses of T cells as well as the prominent accumulation of MDSCs and the marked productions of immunosuppressive mediators in tumor tissues. Thus, our results suggest that CD11c^hi^ DCs are required for the initiation and regulation of anti-tumor T-cell responses as well as the formation of immunogenic TME for the inhibition of the progression of tumors.

Analysis of CD11c:DTA mice revealed that the congenital deficiency of CD11c^hi^ DCs caused the abnormal composition of leukocytes in lymphoid tissues correlated with the enhanced proportions of B cells, PMNs, and monocytes/macrophages under homeostatic and tumor-bearing conditions. Flt3L is reportedly important for the regulation of the development of leukocytes (26–28). We have previously reported that the serum production of Flt3L was enhanced in CD11c:DTA mice under homeostatic conditions, suggesting that cDC lineage is responsible for the major consumption of Flt3L (14). However, we showed that the inhibitor of Flt3 suppressed the enhanced proportions of PMNs and monocytes/macrophages in lymphoid tissues in CD11c:DTA mice. Thus, the secretion of an excess amount of Flt3L in the periphery under the constitutive loss of CD11c^hi^ DCs could lead to the abnormal composition of leukocytes in lymphoid tissues, and that may affect the formation of the pathophysiological immune responses.

Given the decreased proportion of NK cells under the transient depletion of CD11c^+^ DCs (50), CD11c^+^ DCs could be required to maintain NK cell homeostasis through the production of IL-15 (51, 52). However, it has been also shown that IL-2 favors the rapid proliferation of short-lived effector phenotypes of CD8^+^ T cells and NK cells, while IL-15 has its own unique effects on supporting the maintenance of their long-lived memory phenotypes (53). We showed that CD11c:DTA mice exhibited normal proportions of CD3^−^CD19^−^NK1.1^+^NKp46^+^CD49a^−^CD49b^+^ NK cells. Furthermore, they exhibited the enhanced or reduced productions of serum IL-2 or IL-15. Different from the direct linking of the impaired production of IL-15 under the transient depletion of CD11c^+^ DCs with a reduced population size of NK cells, our results suggest that the excessive production of IL-2 compensates for the diminished production of IL-15 for maintaining the population size of CD3^−^CD19^−^NK1.1^+^NKp46^+^CD49a^−^CD49b^+^ NK cells under the constitutive loss of CD11c^hi^ DCs.

CD11c:DTA mice showed a reduced constituency of CD3^−^CD19^−^CD127^+^RORγt^+^ ILC3s in LNs and Spl, although they did not show an expression of CD11c. Furthermore, they exhibited a reduced production of serum IL-1β. Given that IL-1β produced by DCs plays a relevant role in the proliferation of ILC3s (54), a reduced constituency of CD3^−^CD19^−^CD127^+^RORγt^+^ ILC3s in lymphoid tissues may be due to the constitutive loss of CD11c^hi^ DCs as sources of IL-1β rather than the direct toxicity of DTA under the control of the expression of CD11c.

While the transient depletion of cDC1 and cDC2 in zDC (Zbtb46)-DTR mice or CD11c^+^ DCs in CD11c-DTR mice reportedly reduced the effect of tumor vaccination to enhance the survival rates of tumor-bearing mice and to inhibit the development of tumor (55, 56), their transient depletion had little or no effect on the tumor growth (56). In contrast, the constitutive absence of cDC1 in Batf3-deficient mice not only enhanced the progression of tumors but also abrogated the effect of tumor vaccination to inhibit the growth of tumors (9, 57). We showed that the congenital deficiency of CD11c^hi^ DCs in CD11c:DTA mice caused the marked progression of tumor. The reason why the constitutive absence of CD11c^hi^ DCs in CD11c:DTA mice or cDC1 in Batf3-deficient mice leads to the enhanced progression of tumor may be due to the failure of the establishment of the tumor immunosurveillance associated with the occurrence of anti-tumor-specific T_eff_ cells. Indeed, we showed that the constitutive absence of CD11c^hi^ DCs showed a marked reduction of MHC I-OVA pentamer^+^CD44^high^CD8^+^ T cells in tumor tissues and lymphoid tissues. However, CD11c-DTR mice or zDC (Zbtb46)-DTR mice reportedly exhibited the short-term ablation of CD11c^+^ DCs or cDC1 and cDC2 and their rapid recovery following DT injection. Therefore, the transient depletion of CD11c^+^ DCs in CD11c-DTR mice or cDC1 and cDC2 in zDC (Zbtb46)-DTR mice may be insufficient for the induction of the breakdown of the tumor immunosurveillance to enhance the development of tumor, whereas their transient depletion could be sufficient for impairment of the enhanced anti-tumor immune response triggered by tumor vaccination.

In contrast to the published reports (55, 56), analysis of tumor-bearing zDC-DTR mice showed that the transient depletion of cDC1 and cDC2 resulted in the delayed growth of tumors and the enhanced survival rates possibly due to the drastic reduction of CD4^+^Foxp3^+^ T_reg_ cells and maintenance of T_H_ cells in TdLNs (50). However, tumor-bearing CD11c:DTA mice exhibited a reduced proportion of CD4^+^Foxp3^+^ T_reg_ cells and T_eff_ cells in TdLNs. Therefore, the influence of the deficiency of DCs on the population sizes of CD4^+^Foxp3^+^ T_reg_ cells and T_eff_ cells may regulate the direction of the adaptive anti-tumor immune responses that control the development of tumors.

The constitutive elimination of CD11c^hi^ DCs exhibited a more potent enhancement of the growth of the immunogenic tumor than that of the poor immunogenic tumor. Therefore, the impact of the immunogenic function of CD11c^hi^ DCs on adaptive anti-tumor immunity could be linked with the immunogenicity of tumors. However, the TME-mediated modulation of tumor-infiltrated DCs is suggested to impair their function in enhancing the adaptive anti-tumor immunity, thereby promoting the development of tumors (4, 58). While DCs could have the potential to initiate anti-tumor immune responses by recruiting and activating various types of immune cells in TdLNs, the TME could secrete immunosuppressive mediators that limit the immunogenic function of DCs and instead skew DCs to a tolerogenic function.

Previous studies have shown that CD11c:DTA mice had an unimpaired compartment of T cells in peripheral lymphoid tissues, while they exhibited myeloproliferative disorder (MPD) with lymphadenopathies (13). However, we showed that CD11c:DTA mice displayed a reduced constituency of T cells in LNs and Spl, whereas they did not develop MPD with normal sizes of LNs and Spl. Although the precise reason for these apparent discrepancies remains unclear, the different environmental conditions may affect the immune cell homeostasis.

The congenital deficiency of CD11c^hi^ DCs not only reduced the expression of CD44 on CD4^+^ T cells and CD8^+^ T cells in lymphoid tissues under homeostatic and tumor-bearing conditions but also enhanced their expressions of several immune checkpoint molecules under tumor-bearing conditions. Taken together, these results suggest that CD11c^hi^ DCs are required to control the activation status of T cells in lymphoid tissues under homeostatic and tumor-bearing conditions.

We showed that the congenital deficiency of CD11c^hi^ DCs enhanced the proportion of tumor-infiltrating CD4^+^ T_EM_ cells, while it reduced their expressions of certain immune checkpoint molecules. However, the constitutive ablation of CD11c^hi^ DCs had little or no effect on the proportion of tumor-infiltrating CD8^+^ T_EM_ cells and their expressions of the immune checkpoint molecules. Thus, these results suggest that CD11c^hi^ DCs control the manifestation of the effector memory phenotype of tumor-infiltrating CD4^+^ T cells and their expressions of the immune checkpoint molecules.

We showed that the constitutive deficiency of CD11c^hi^ DCs caused the almost complete inhibition of the priming of Ag-specific CD4^+^ T cells in LNs and TdLNs under homeostatic conditions with soluble antigenic immunization and tumor-bearing conditions. Therefore, these results suggest that CD11c^hi^ DCs are critical APCs to present soluble and tumor cell-associated Ags to CD4^+^ T cells for their Ag-specific priming.

The constitutive absence of CD11c^hi^ DCs partially inhibited the Ag-specific priming of CD8^+^ T cells in LNs under homeostatic conditions with soluble antigenic immunization. It has been reported that pDCs could prime Ag-specific CD8^+^ T cells through cross-presentation (59–61). Since pDCs were partially depleted in lymphoid tissues of CD11c:DTA mice, it is possible that residual pDCs as well as other APCs, including monocytes/macrophages, participate in cross-priming of CD8^+^ T cells with soluble Ag.

We showed that the congenital deficiency of CD11c^hi^ DCs not only suppressed Ag-specific priming of CD8^+^ T cells in TdLNs and tumor tissues but also abolished the generation of Ag-specific CTLs in lymphoid and tumor tissues under tumor-bearing conditions. Thus, our findings suggest that CD11c^hi^ DCs are the most potent APCs to present tumor cell-associated Ag to CD8^+^ T cells through cross-presentation for their Ag-specific priming to generate tumor-specific CTLs. Previous studies have shown that the transient depletion of CD11c^+^ DCs, including cDC1, cDC2, and Mo-DCs/DC3, inhibited Ag-specific priming of CD8^+^ T cells in tumor tissues (62). However, it has been shown that Ag-specific priming of CD8^+^ T cells was reduced in TdLNs, whereas their response was not affected in tumor tissues under the transient depletion of cDC1 and cDC2, but not Mo-DCs/DC3 (30). Furthermore, Mo-DCs/DC3 have been shown to cross-prime Ag-specific CD8^+^ T cells (30). Collectively, these phenomena imply that Mo-DCs/DC3 play a key role in the cross-priming of CD8^+^ T cells in tumor tissues, while cDC1 and cDC2 are essential for this response in TdLNs.

Similar to the published reports (14, 63), the congenital deficiency of CD11c^hi^ DCs reduced the proportion of CD4^+^Foxp3^+^ T_reg_ cells in lymphoid tissues under homeostatic and tumor-bearing conditions, suggesting the importance of CD11c^hi^ DCs in the maintenance of the population size of CD4^+^Foxp3^+^ T_reg_ cells. Conversely, the constitutive absence of CD11c^hi^ DCs enhanced the proportion of CD4^+^Foxp3^+^RORγt^+^ T_reg_ cells in lymphoid tissues under homeostatic and tumor-bearing conditions. It has been reported that CD4^+^Foxp3^+^RORγt^+^ T_reg_ cells, possibly derived from CD4^+^Foxp3^+^RORγt^−^ T_reg_ cells, had a prominent suppressive activity against the responses of CD4^+^ T_eff_ cells (45, 46). Furthermore, the intestinal CD4^+^Foxp3^+^RORγt^+^ T_reg_ cells induced by gut microbiota have been shown to contribute to the suppression of gut inflammation (64, 65), the inhibition of T_H_2-driven defense against helminths and T_H_2-associated colitis (66), and the inhibition of intestinal T_H_2-associated allergy (67). While the development of CD4^+^Foxp3^+^RORγt^+^ T_reg_ cells and their role in the regulation of anti-tumor immunity as well as their relation to the intestinal CD4^+^Foxp3^+^RORγt^−^ T_reg_ cells remain unclear, it is possible that CD4^+^Foxp3^+^RORγt^−^ T_reg_ cells convert into CD4^+^Foxp3^+^RORγt^+^ T_reg_ cells in the milieu of the abundance of various proinflammatory cytokines under the constitutive absence of CD11c^hi^ DCs (14), and they suppress anti-tumor functions of CD4^+^ T_eff_ cells and CTLs in lymphoid tissues.

Previous studies have reported that tumor cells and immune cells as well as fibroblasts in the tumor stroma could produce a variety of inflammatory mediators, including myeloid growth factors and proinflammatory cytokines, leading to the creation of autocrine and paracrine loops for the generation of the chronic inflammation in the TME (41, 68). Furthermore, the chronic inflammation in the TME could contribute to the generation of MDSCs, possibly due to the occurrence of the aberrant myelopoiesis to skew the differentiation of PMNs and monocytes/macrophages in favor of MDSCs (41, 68). However, we have previously reported that the congenital deficiency of CD11c^hi^ DCs triggers spontaneous systemic inflammation with the massive productions of various pro-inflammatory cytokines and Flt3L, accompanied by the abnormal composition of leukocytes, including the enhanced generation of PMNs, in lymphoid tissues (14). Furthermore, we showed that the inhibitor of Flt3 suppressed the enhanced generation of PMNs and monocytes/macrophages in lymphoid tissues in CD11c:DTA mice. Thus, these phenomena imply that the constitutive loss of CD11c^hi^ DCs triggers the massive secretion of Flt3L in the periphery and the chronic inflammation in the TME, which enhances the generation of MDSCs from myeloid cells in the context of the aberrant myelopoiesis during the development of tumor.

Accumulating lines of evidence suggest that MDSCs play a critical role in the development of tumors (40–42). We showed that the elimination of MDSCs exhibited a more potent reduction of tumor growth in tumor-bearing CD11c:DTA mice than in tumor-bearing WT mice. Thus, these results suggest that the promoted generation of MDSCs and their accumulation in the TME contribute to the enhanced progression of tumors under the constitutive loss of CD11c^hi^ DCs.

Having demonstrated the linking of the constitutive deficiency of CD11c^hi^ DCs with the marked accumulation of MDSCs in tumor tissues, tumor-infiltrating MDSCs could contribute to the enhanced secretions of several immunosuppressive cytokines and metabolite-generating enzymes in tumor tissues under the constitutive ablation of CD11c^hi^ DCs. Thus, our findings suggest that the congenital deficiency of CD11c^hi^ DCs promotes the emergence of MDSCs in tumor tissues and facilitates the formation of the immunosuppressive TME through the production of immunosuppressive mediators. Taken together, CD11c^hi^ DCs could be crucial for generating the immunogenic TME by attenuating the generation of MDSCs and their accumulation in tumor tissues for the suppression of the development of tumors.

In conclusion, our results suggest that the congenital deficiency of CD11c^hi^ DCs leads to the impaired responses of tumor-specific T_eff_ cells and the occurrence of the immunosuppressive TME, possibly mediated through the marked accumulation of MDSCs and their prominent productions of immunosuppressive mediators, and that results in the acceleration of the development of tumor. Thus, our findings unravel the importance of CD11c^hi^ DCs in the protection from the progression of tumors by generating anti-tumor T_eff_-cell responses and immunogenic TME. A better understanding of the nature of CD11c^hi^ DCs and the manipulation to enforce their function may open new avenues for exploring novel tumor-specific immunotherapeutic treatments.

## Data Availability

The original contributions presented in the study are included in the article/**Supplementary Material**. Further inquiries can be directed to the corresponding author.
