# Supplementary material for: Crucial role of dendritic cells in the generation of anti-tumor T-cell responses and immunogenic tumor microenvironment to suppress tumor development
Source: Front Immunol. 2024 Aug 14;15:1200461. doi: 10.3389/fimmu.2024.1200461 (PMC11349553; doi:10.3389/fimmu.2024.1200461)
Supplement: Supplementary file 1 [file DataSheet1.pdf]

Supplementary Material

Crucial role of dendritic cells in the generation of anti-tumor T-cell responses and immunogenic tumor microenvironment to suppress tumor development

Moe Tominaga, Tomofumi Uto, Tomohiro Fukaya, Shuya Mitoma, Dieter Riethmacher, Kunihiko Umekita, Yoshihiro Yamashita and Katsuaki Sato*

***Correspondence:** Katsuaki Sato: katsuaki_sato@med.miyazaki-u.ac.jp

TABLE S1. List of fluorescein-conjugated mAb

Name	Supplier	clone	conjugate
CD3ε	BD	145-2C11	FITC,APC,BV421,BV510
CD3	Biolegend	17A2	PE-Cy7, BV421
CD4	BD	RM4-5	PE, PE-Cy7
CD8α	BD	53.6.7	FITC, PE, APC-Cy7
CD8	MBL	KT15	FITC
CD11b	BD	M1/70	BV510
CD11c	BD	HL3	PE,PE-Cy7, BV421
CD19	BD	1D3	PE
CD19	Biolegend	6D5	BV510
CD44	BD	1M7	BV510
CD45	Biolegend	I3/2.3	FITC
CD45R/B220	Biolegend	RA3-6B2	FITC,PE-Cy7, APC-Cy7
CD45.1	BD	A20	FITC
CD49a	Biolegend	HMa1	PE
CD49b	Biolegend	DX5	FITC
CD62L	BD	MEL-14	BV421
CD64	Biolegend	X54-5/7.1	PE-Cy7
CD80	Biolegend	16-10A1	PE
CD127	Biolegend	A7R34	FITC
CD206	Biolegend	C068C2	FITC
CD207	Biolegend	4C7	APC
F4/80	Biolegend	BM8	APC,BV421
Foxp3	eBioscience	FJK-16s	APC
Gr-1	Biolegend	RB6-8C5	biotin, FITC
H-2Kb OVA peptide pentamer	Proimmune	-	APC
H-2Db gp100 peptide tetramer	MBL	-	APC
LAG-3	Biolegend	C9B7W	PE
MHC class II (I-A/I-E)	Biolegend	M5/114.15.2	BV510
NK1.1	BD	PK136	FITC,APC
NKp46	Biolegend	29A1.4	APC-Cy7
PD-1	Biolegend	RMP1-30	PE
RORγt	BD	Q31-378	PE, BV421
Siglec-H	Biolegend	551	PE
SIRPα	Biolegend	P84	PE
TIM-3	Biolegend	RMT3-23	PE
Vα2-TCR	eBioscience	B20.1	APC
XCR1	Biolegend	ZET	FITC

TABLE S2. List of RT-qPCR primers

Primer	Sequence
gapdh-F	AAATTCAACGGCACAGTCAAG
gapdh-R	TGGTGGTGAAGACACCAGTAG
il10-F	TGCAGCAGCTCAGAGGGTT
il-10-R	TGGCCACAGTTTTCAGGGAT
tgfb-F	ACCATGCCAACTTCTGTCTG
tgfb-R	CGGGTTGT GTTGGTTGTAGA
ido-F	ACTGTGTCCTGGCAAACTGGAAG
ido-R	AAGCTGCGATTTCCACCAATAGAG
vegf-F	AATGCTTTCTCCGCTCTGAA
vegf-R	GCTTCCTACAGCACAGCAGA
arginase-F	AAGACAGCAGAGGAGGTGAAG
arginase-R	TAGTCAGTCCCTGGCTTATGG
inos-F	AACAATTCCTGGCGTTACCTT
inos-R	TGTATTCCGTCTCCTTGGTTC
cox2-F	TGGGTGTGAAGGGAAATAAGG
cox2-R	CATCATATTTGAGCCTTGGGG
mpges-F	AGGATGCGCTGAAACGTGGAG
mpges-R	CCGAGGAAGAGGAAAGGATAG

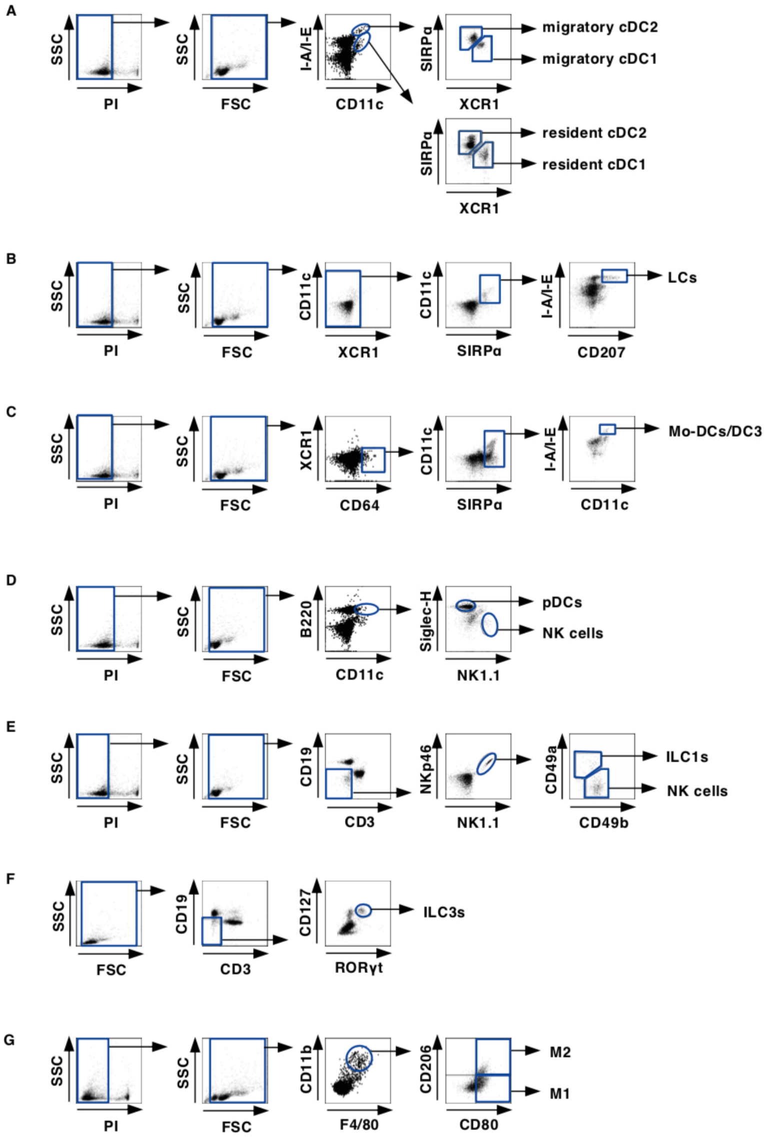

Supplementary Figure 1. Gating strategy in flow cytometry to identify leukocytes in LNs in WT mice under homeostatic conditions. (**A**) Leukocytes in LNs were analyzed in the indicated sequential gates for propidium iodide (PI)-side scatter (SSC) to exclude dead cells, and forward scatter (FSC)-SSC to identify live leukocytes. Live leukocytes were analyzed in the indicated gates for CD11c-I-A/I-E to identify I-A/I-E^{hi}CD11c^{+} migratory cDCs and I-A/I-E^{+}CD11c^{+} resident cDCs, and they were further analyzed in the indicated gates for XCR1-SIRPα to identify I-A/I-E^{hi}CD11c^{+}XCR1^{+}SIRPα^{-} migratory cDC1, I-A/I-E^{hi}CD11c^{+}XCR1^{-}SIRPα^{+} migratory cDC2, I-A/I-E^{+}CD11c^{+}XCR1^{+}SIRPα^{-} resident cDC1, and I-A/I-E^{+}CD11c^{+}XCR1^{-}SIRPα^{+} resident cDC2. (**B**) Leukocytes in LNs were analyzed in the indicated sequential gates for PI-SSC to exclude dead cells, and FSC-SSC to identify live leukocytes. Live leukocytes were analyzed in the indicated gates for XCR1-CD11c to identify CD11c^{+}XCR1^{-} leukocytes followed by the indicated gates for SIRPα-CD11c to identify CD11c^{+}XCR1^{-}SIRPα^{+} leukocytes, and they were further analyzed in the indicated gates for CD207-I-A/I-E to identify I-A/I-E^{hi}CD11c^{+}CD207^{+}XCR1^{-}SIRPα^{+} LCs. (**C**) Leukocytes in LNs were analyzed in the indicated sequential gates for PI-SSC to exclude dead cells, and FSC-SSC to identify live leukocytes. Live leukocytes were analyzed in the indicated gates for CD64-XCR1 to identify

XCR1^{-}CD64^{+} leukocytes followed by the indicated gates for SIRPα-CD11c to identify CD11c^{+}XCR1^{-}SIRPα^{+}CD64^{+} leukocytes, and they were further analyzed in the indicated gates for CD11c-I-A/I-E to identify I-A/I-E^{hi}CD11c^{+}XCR1^{-}SIRPα^{+}CD64^{+} Mo-DCs/DC3. (**D**) Leukocytes in LNs were analyzed in the indicated sequential gates for PI-SSC to exclude dead cells, and FSC-SSC to identify live leukocytes. Live leukocytes were analyzed in the indicated gates for CD11c-B220 to identify CD11c^{+}B220^{+} leukocytes, and they were further analyzed in the indicated gates for NK1.1-Siglec-H to identify CD11c^{+}B220^{+}Siglec-H^{+}NK1.1^{-} pDCs and CD11c^{+}B220^{+}Siglec-H^{-}NK1.1^{+} NK cells. (**E**) Leukocytes in LNs were analyzed in the indicated sequential gates for PI-SSC to exclude dead cells, and FSC-SSC to identify live leukocytes. Live leukocytes were analyzed in the indicated gates for CD3-CD19 to identify CD3^{-}CD19^{-} leukocytes followed by the indicated gates for NK1.1-NKp46 to identify CD3^{-}CD19^{-}NK1.1^{+}NKp46^{+} leukocytes, and they were further analyzed in the indicated gates for CD49b-CD49a to identify CD3^{-}CD19^{-}NK1.1^{+}NKp46^{+}CD49a^{-}CD49b^{+} NK cells and CD3^{-}CD19^{-}NK1.1^{+}NKp46^{+}CD49a^{+}CD49b^{-} ILC1s. (**F**) Leukocytes in LNs were analyzed in the indicated sequential gates for FSC-SSC to identify leukocytes. Leukocytes were analyzed in the indicated gates for CD3-CD19 to identify CD3^{-}CD19^{-} leukocytes, and they were further analyzed in the indicated gates for RORγt-CD127 to identify CD3^{-}CD19^{-}CD127^{+}RORγt^{+} ILC3s. (**G**) Leukocytes in LNs were analyzed in the indicated sequential gates for PI-SSC to exclude dead cells, and FSC-SSC to identify live leukocytes. Live leukocytes were analyzed in the indicated gates for F4/80-CD11b to identify CD11b^{+}F4/80^{+} leukocytes, and they were further analyzed in the indicated gates for CD80-CD206 to identify CD11b^{+}F4/80^{+}CD80^{+} M1-like macrophages and CD11b^{+}F4/80^{+}CD206^{+} M2-like macrophages. All data are representative of at least three independent experiments.

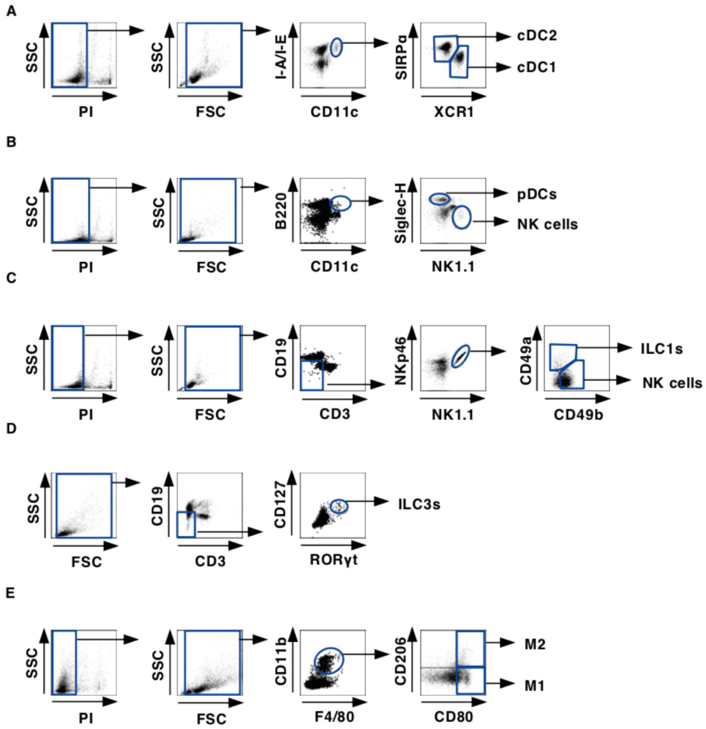

Supplementary Figure 2. Gating strategy in flow cytometry to identify leukocytes in Spl in WT mice under homeostatic conditions. (**A**) Leukocytes in Spl were analyzed in the indicated sequential gates for PI-SSC to exclude dead cells, and FSC-SSC to identify live leukocytes. Live leukocytes were analyzed in the indicated gates for CD11c-I-A/I-E to identify I-A/I-E^{+}CD11c^{+} cDCs, and they were further analyzed in the indicated gates for XCR1-SIRPα to identify I-A/I-E^{+}CD11c^{+}XCR1^{+}SIRPα^{-} cDC1 and I-A/I-E^{+}CD11c^{+}XCR1^{-}SIRPα^{+} cDC2. (**B**) Leukocytes in Spl were analyzed in the indicated sequential gates for PI-SSC to exclude dead cells, and FSC-SSC to identify live leukocytes. Live leukocytes were analyzed in the indicated gates for CD11c-B220 to identify CD11c^{+}B220^{+} leukocytes, and they were further analyzed in the indicated gates for NK1.1-Siglec-H to identify CD11c^{+}B220^{+}Siglec-H^{+}NK1.1^{-} pDCs and CD11c^{+}B220^{+}Siglec-H^{-}NK1.1^{+} NK cells. (**C**) Leukocytes in Spl were analyzed in the indicated sequential gates for PI-SSC to exclude dead cells, and FSC-SSC to identify live leukocytes. Live leukocytes were analyzed in the indicated gates for CD3-CD19 to identify CD3^{-}CD19^{-} leukocytes followed by the indicated gates for NK1.1-NKp46 to identify CD3^{-}CD19^{-}NK1.1^{+}NKp46^{+} leukocytes, and they were further analyzed in the indicated gates for CD49b-CD49a to identify CD3^{-}CD19^{-}NK1.1^{+}NKp46^{+}CD49a^{-}CD49b^{+} NK cells and CD3^{-}CD19^{-}NK1.1^{+}NKp46^{+}CD49a^{+}CD49b^{-} ILC1s. (**D**) Leukocytes in Spl were analyzed in the indicated sequential gates for FSC-SSC to identify leukocytes. Leukocytes were analyzed in the indicated gates for CD3-CD19 to identify CD3^{-}CD19^{-} leukocytes, and they were further analyzed in the indicated gates for RORγt-CD127 to identify CD3^{-}CD19^{-}CD127^{+}RORγt^{+} ILC3s. (**E**) Leukocytes in Spl were analyzed in the indicated sequential gates for PI-SSC to exclude dead cells, and FSC-SSC to identify live leukocytes. Live leukocytes were analyzed in the indicated gates for F4/80-CD11b to identify CD11b^{+}F4/80^{+} leukocytes, and they were further analyzed in the indicated gates for CD80-CD206 to identify CD11b^{+}F4/80^{+}CD80^{+} M1-like macrophages and CD11b^{+}F4/80^{+}CD206^{+} M2-like macrophages. All data are representative of at least three independent experiments.

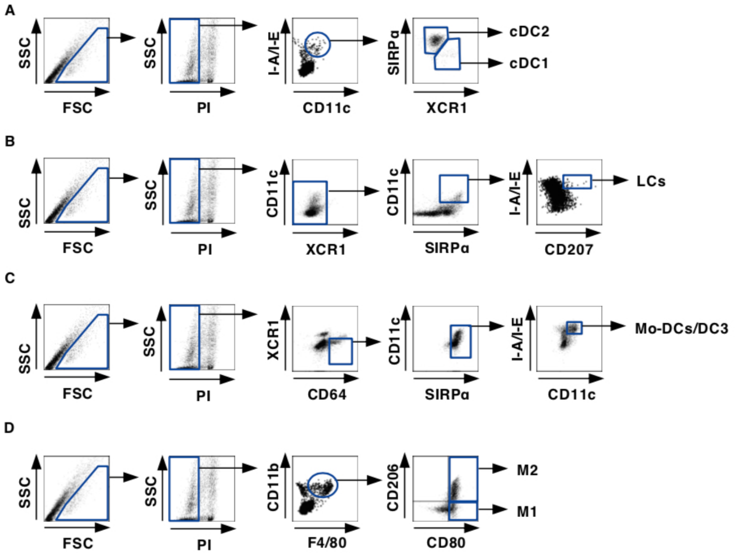

Supplementary Figure 3. Gating strategy in flow cytometry to identify leukocytes in tumor tissues in WT mice under tumor-bearing condition. (**A**) CD45$^+$ leukocytes in tumor tissues were analyzed in the indicated sequential gates for FSC-SSC to identify leukocytes, and PI-SSC to exclude dead cells and identify live leukocytes. Live leukocytes were analyzed in the indicated gates for CD11c-I-A/I-E to identify I-A/I-E^{hi}CD11c$^+$ migratory cDCs, and they were further analyzed in the indicated gates for XCR1-SIRPα to identify I-A/I-E^{hi}CD11c$^+$XCR1$^+$SIRPα$^-$ migratory cDC1 and I-A/I-E^{hi}CD11c$^+$XCR1$^-$SIRPα$^+$ migratory cDC2. (**B**) CD45$^+$ leukocytes in tumor tissues were analyzed in the indicated sequential gates for FSC-SSC to identify leukocytes, and PI-SSC to exclude dead cells and identify live leukocytes. Live leukocytes were analyzed in the indicated gates for XCR1-CD11c to identify CD11c$^+$XCR1$^-$ leukocytes followed by the indicated gates for SIRPα-CD11c to identify CD11c$^+$XCR1$^-$SIRPα$^+$ leukocytes, and they were further analyzed in the indicated gates for CD207-I-A/I-E to identify I-A/I-E^{hi}CD11c$^+$CD207$^+$XCR1$^-$SIRPα$^+$ LCs. (**C**) CD45$^+$ leukocytes in tumor tissues were analyzed in the indicated sequential gates for FSC-SSC to identify leukocytes, and PI-SSC to exclude dead cells and identify live leukocytes. Live leukocytes were analyzed in the indicated gates for CD64-XCR1 to identify XCR1$^-$CD64$^+$ leukocytes followed by the indicated gates for SIRPα-CD11c to identify CD11c$^+$XCR1$^-$SIRPα$^+$CD64$^+$ leukocytes, and they were further analyzed in the indicated gates for CD11c-I-A/I-E to identify I-A/I-E^{hi}CD11c$^+$XCR1$^-$SIRPα$^+$CD64$^+$ Mo-DCs/DC3. (**D**) CD45$^+$ leukocytes in tumor tissues were analyzed in the indicated sequential gates for FSC-SSC to identify leukocytes, and PI-SSC to exclude dead cells and identify live leukocytes. Live leukocytes were analyzed in the indicated gates for F4/80-CD11b to identify CD11b$^+$F4/80$^+$ leukocytes, and they were further analyzed in the indicated gates for CD80-CD206 to identify CD11b$^+$F4/80$^+$CD80$^+$ M1-like macrophages and CD11b$^+$F4/80$^+$CD206$^+$ M2-like macrophages. All data are representative of at least three independent experiments.

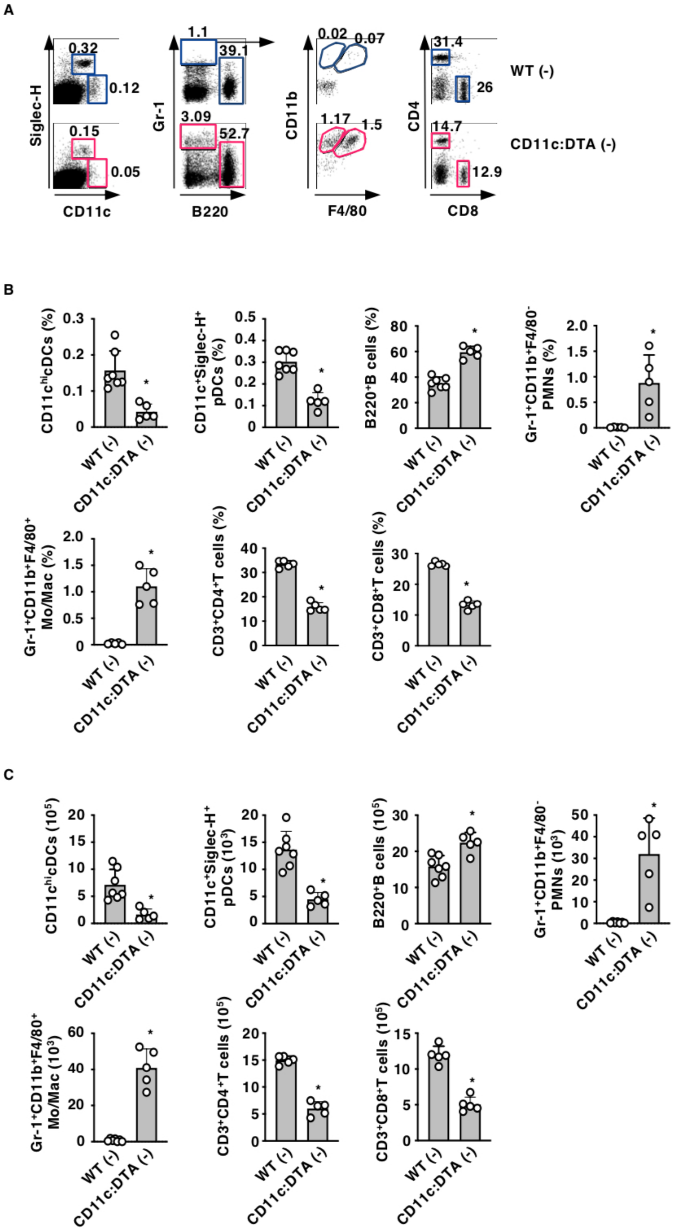

Supplementary Figure 4. Deficiency of CD11c^{hi} DCs influences the cellularity in LNs under homeostatic conditions. Cell surface expression profile **(A)**, proportion **(B)**, and absolute number **(C)** of leukocytes in LNs. Data are obtained from five to seven individual samples in a single experiment. Numbers in the dot plot represent the proportion of the indicated cell populations among leukocytes. *$P < 0.05$ compared with WT mice by two-sided unpaired Student's *t*-test. All data are representative of at least three independent experiments.

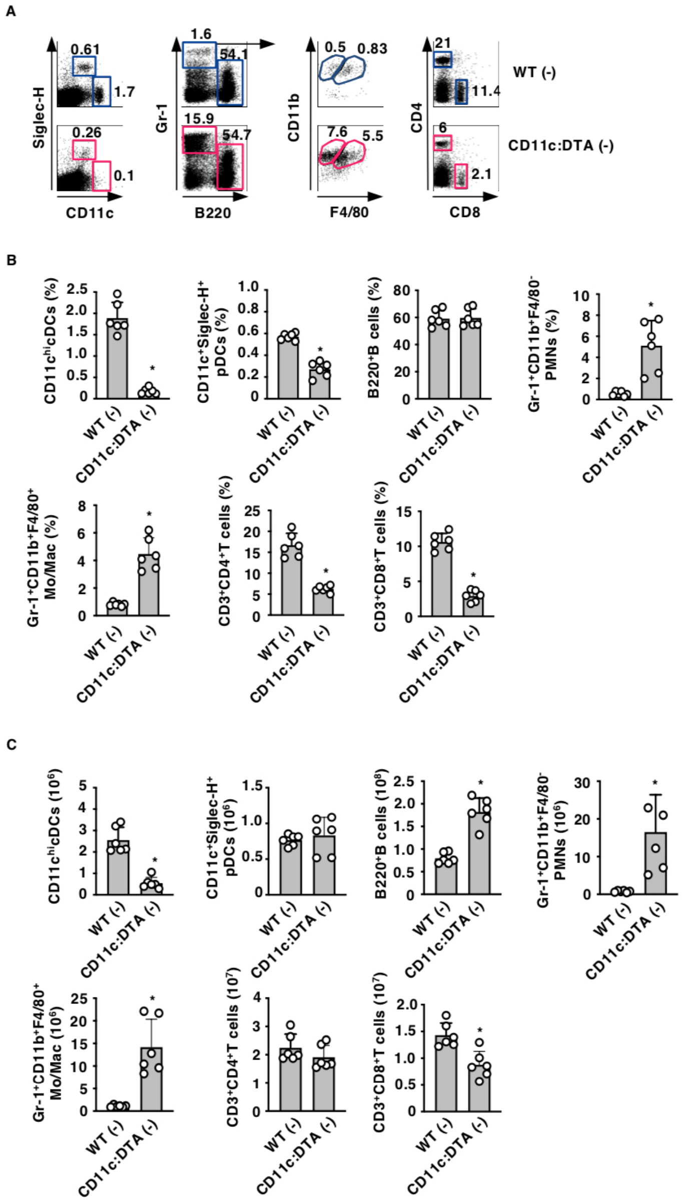

Supplementary Figure 5. Deficiency of CD11c^{hi} DCs influences the cellularity in Spl under homeostatic conditions. Cell surface expression profile **(A)**, proportion **(B)**, and absolute number **(C)** of leukocytes in Spl. Data are obtained from five to six individual samples in a single experiment. Numbers in the dot plot represent the proportion of the indicated cell populations among leukocytes. *$P < 0.05$ compared with WT mice by two-sided unpaired Student's *t*-test. All data are representative of at least three independent experiments.

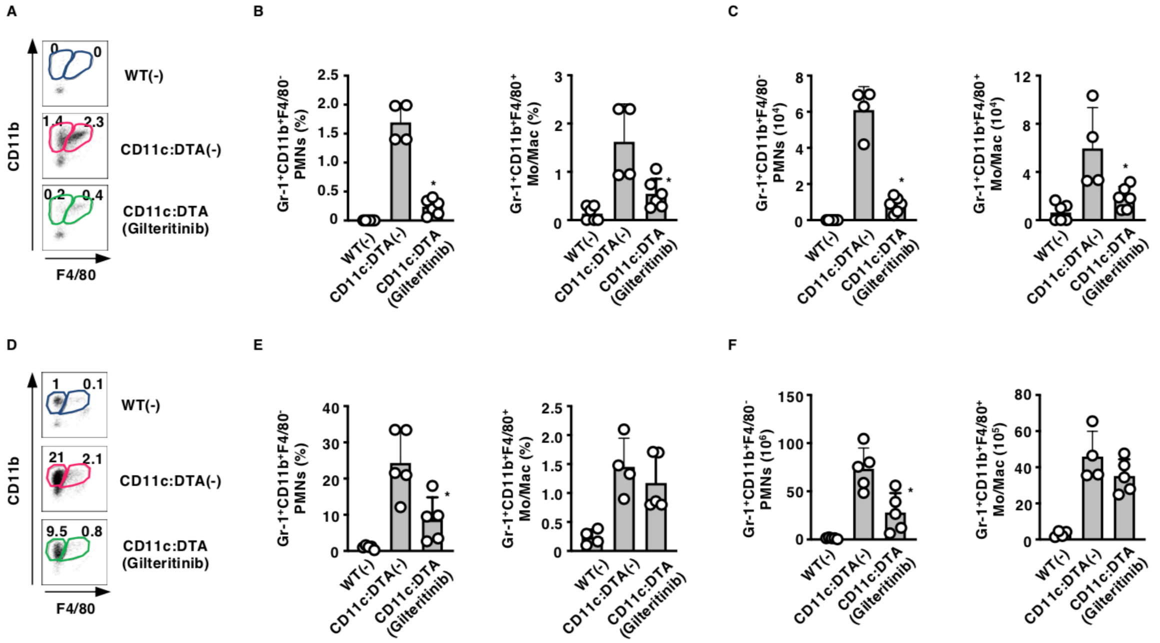

Supplementary Figure 6. Role of Flt3L in the enhanced generations of PMNs and monocytes/macrophages under the deficiency of CD11c^{hi} DCs. CD11c:DTA mice were daily treated with or without Gilteritinib for 20 days. Cell surface expression profile **(A, D)**, proportion **(B, E)**, and absolute number **(C, F)** of Gr-1^{+}CD11b^{+}F4/80^{-} PMNs and Gr-1^{+}CD11b^{+}F4/80^{+} monocytes/macrophages among Gr-1^{+}B220^{-} leukocytes in LNs **(A-C)** and Spl **(D-F)** on 21 days after the start of the administration with Gilteritinib. Data are obtained from four to six individual samples in a single experiment. Numbers in the dot plot represent the proportion of the indicated cell populations among leukocytes. *P < 0.05 compared with untreated CD11c:DTA mice by two-sided unpaired Student's t-test. All data are representative of at least three independent experiments.

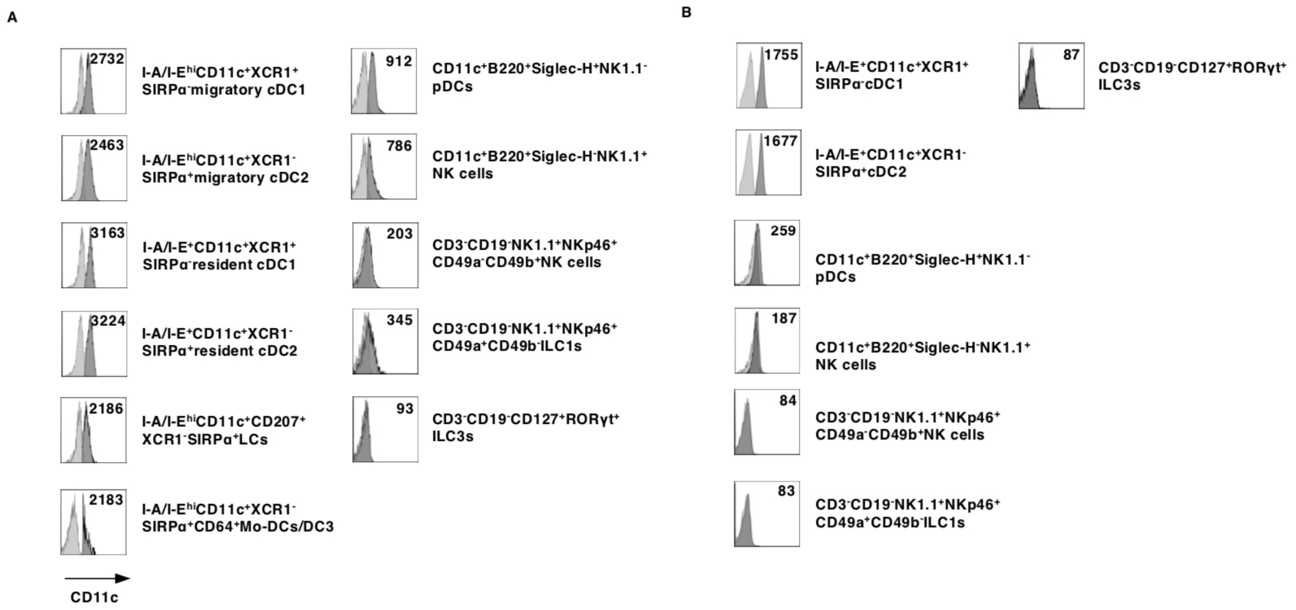

Supplementary Figure 7. Cell surface expression of CD11c on the subsets of leukocytes in LNs and Spl in WT mice under homeostatic conditions. (**A**) Mean fluorescence intensity (MFI) of the cell surface expression of CD11c on I-A/I-E^{hi}CD11c^{+}XCR1^{+}SIRPα^{-} migratory cDC1, I-A/I-E^{hi}CD11c^{+}XCR1^{-}SIRPα^{+} migratory cDC2, I-A/I-E^{+}CD11c^{+}XCR1^{+}SIRPα^{-} resident cDC1, I-A/I-E^{+}CD11c^{+}XCR1^{-}SIRPα^{+} resident cDC2, I-A/I-E^{hi}CD11c^{+}CD207^{+}XCR1^{-}SIRPα^{+} LCs, I-A/I-E^{hi}CD11c^{+}XCR1^{-}SIRPα^{+}CD64^{+} Mo-DCs/DC3, CD11c^{+}B220^{+}Siglec-H^{+}NK1.1^{-} pDCs, CD11c^{+}B220^{+}Siglec-H^{-}NK1.1^{+} NK cells, CD3^{-}CD19^{-}NK1.1^{+}NKp46^{+}CD49a^{-}CD49b^{+} NK cells, CD3^{-}CD19^{-}NK1.1^{+}NKp46^{+}CD49a^{+}CD49b^{-} ILC1s, and CD3^{-}CD19^{-}CD127^{+}RORγt^{+} ILC3s in LNs in WT mice. (**B**) MFI of the cell surface expression of CD11c on I-A/I-E^{+}CD11c^{+}XCR1^{+}SIRPα^{-} cDC1, I-A/I-E^{+}CD11c^{+}XCR1^{-}SIRPα^{+} cDC2, CD11c^{+}B220^{+}Siglec-H^{+}NK1.1^{-} pDCs, CD11c^{+}B220^{+}Siglec-H^{-}NK1.1^{+} NK cells, CD3^{-}CD19^{-}NK1.1^{+}NKp46^{+}CD49a^{-}CD49b^{+} NK cells, CD3^{-}CD19^{-}NK1.1^{+}NKp46^{+}CD49a^{+}CD49b^{-} ILC1s, and CD3^{-}CD19^{-}CD127^{+}RORγt^{+} ILC3s in Spl in WT mice. All data are representative of at least three independent experiments.

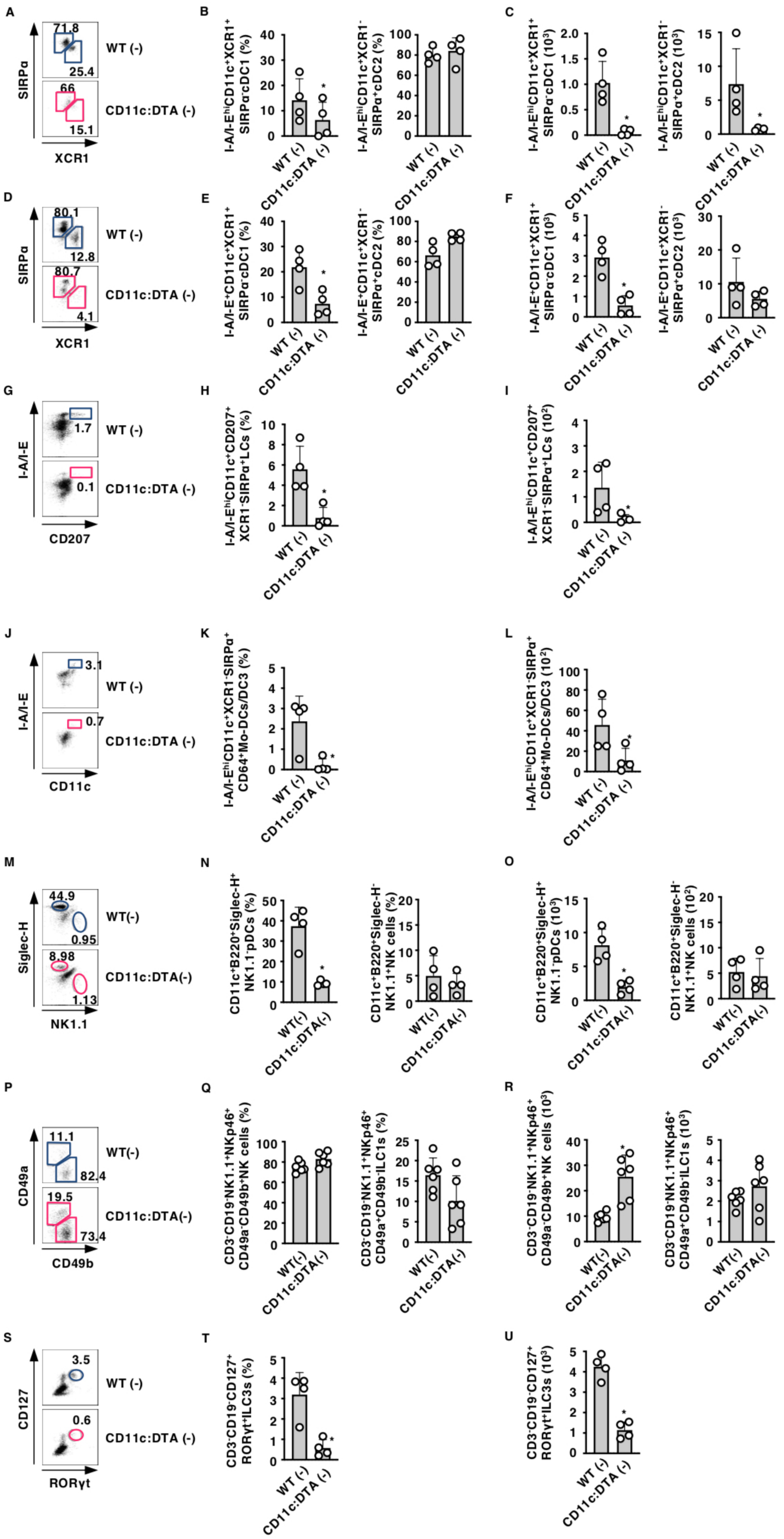
A
SIRPα
XCR1
WT (-)
CD11c:DTA (-)
B
I-A/I-E^hi CD11c^+ XCR1^+ SIRPα^- cDC1 (%)
I-A/I-E^hi CD11c^+ XCR1^- SIRPα^+ cDC2 (%)
C
I-A/I-E^hi CD11c^+ XCR1^+ SIRPα^- cDC1 (10^3)
I-A/I-E^hi CD11c^+ XCR1^- SIRPα^+ cDC2 (10^3)
D
E
I-A/I-E^+ CD11c^+ XCR1^+ SIRPα^- cDC1 (%)
I-A/I-E^+ CD11c^+ XCR1^- SIRPα^+ cDC2 (%)
F
I-A/I-E^+ CD11c^+ XCR1^+ SIRPα^- cDC1 (10^3)
I-A/I-E^+ CD11c^+ XCR1^- SIRPα^+ cDC2 (10^3)
G
I-A/I-E
CD207
H
I-A/I-E^hi CD11c^+ CD207^+ XCR1^- SIRPα^+ LCs (%)
I
I-A/I-E^hi CD11c^+ CD207^+ XCR1^- SIRPα^+ LCs (10^2)
J
CD11c
K
I-A/I-E^hi CD11c^+ XCR1^- SIRPα^+ CD64^+ Mo-DCs/DC3 (%)
L
I-A/I-E^hi CD11c^+ XCR1^- SIRPα^+ CD64^+ Mo-DCs/DC3 (10^2)
M
Siglec-H
NK1.1
WT(-)
CD11c:DTA(-)
N
CD11c^+ B220^+ Siglec-H^+ NK1.1^- pDCs (%)
CD11c^+ B220^+ Siglec-H^- NK1.1^+ NK cells (%)
O
CD11c^+ B220^+ Siglec-H^+ NK1.1^- pDCs (10^3)
CD11c^+ B220^+ Siglec-H^- NK1.1^+ NK cells (10^2)
P
CD49a
CD49b
Q
CD3^- CD19^- NK1.1^+ NKp46^+ CD49a^- CD49b^+ NK cells (%)
CD3^- CD19^- NK1.1^+ NKp46^+ CD49a^+ CD49b^- ILC1s (%)
R
CD3^- CD19^- NK1.1^+ NKp46^+ CD49a^- CD49b^+ NK cells (10^3)
CD3^- CD19^- NK1.1^+ NKp46^+ CD49a^+ CD49b^- ILC1s (10^3)
S
CD127
RORγt
T
CD3^- CD19^- CD127^+ RORγt^+ ILC3s (%)
U
CD3^- CD19^- CD127^+ RORγt^+ ILC3s (10^3)

Supplementary Figure 8. Deficiency of CD11c^{hi} DCs influences the constituencies of the subsets of leukocytes in LNs under homeostatic conditions. Cell surface expression profile (**A, D, G, J, M, P, S**), proportion (**B, E, H, K, N, Q, T**), and absolute number (**C, F, I, L, O, R, U**) of XCR1$^+$SIRPα$^-$ migratory cDC1 and XCR1$^-$SIRPα$^+$ migratory cDC2 among I-A/I-E^{hi}CD11c$^+$ leukocytes **(A-C)**, XCR1$^+$SIRPα$^-$ resident cDC1 and XCR1$^-$SIRPα$^+$ resident cDC2 among I-A/I-E$^+$CD11c$^+$ leukocytes **(D-F)**, I-A/I-E^{hi}CD207$^+$ LCs among CD11c$^+$XCR1$^-$SIRPα$^+$ leukocytes (**G-I**), I-A/I-E^{hi}CD11c$^+$ Mo-DCs/DC3 among XCR1$^-$ SIRPα$^+$CD64$^+$ leukocytes (**J-L**), Siglec-H$^+$NK1.1$^-$ pDCs and Siglec-H$^-$ NK1.1$^+$ NK cells among CD11c$^+$B220$^+$ leukocytes **(M-O)**, CD49a$^-$CD49b$^+$ NK cells and CD49a$^+$CD49b$^-$ ILC1s among CD3$^-$CD19$^-$NK1.1$^+$NKp46$^+$ leukocytes **(P-R)**, and RORγt$^+$CD127$^+$ ILC3s among CD3$^-$CD19$^-$ leukocytes **(S-U)** in LNs. Data are obtained from four to six individual samples in a single experiment. Numbers in the dot plot represent the proportion of the indicated cell populations among leukocytes. *P < 0.05 compared with WT mice by two-sided unpaired Student's *t*-test. All data are representative of at least three independent experiments.

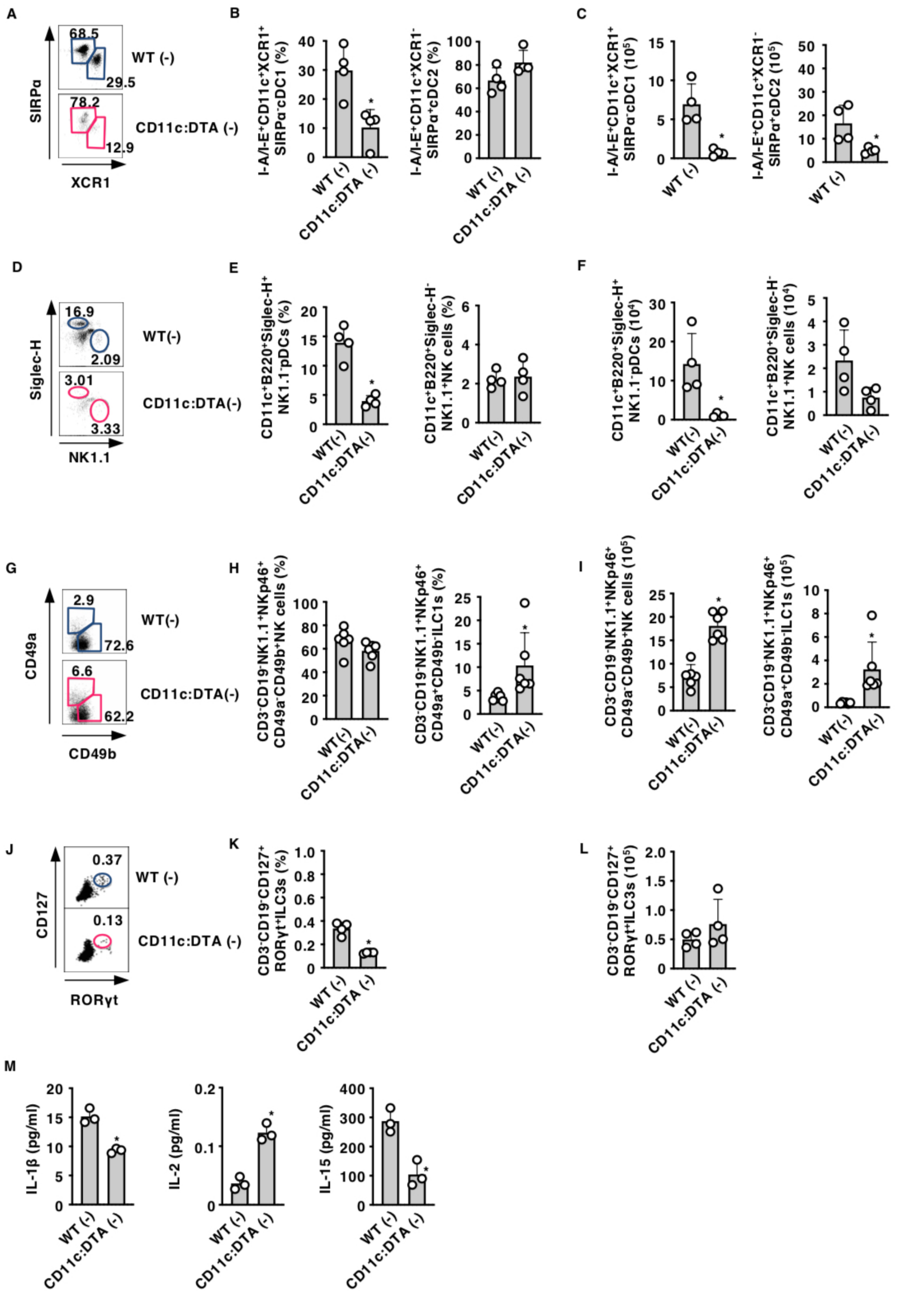

Supplementary Figure 9. Deficiency of CD11c^{hi} DCs influences the constituencies of the subsets of leukocytes in Spl under homeostatic conditions. (**A-L**) Cell surface expression profile (**A, D, G, J**), proportion (**B, E, H, K**), and absolute number (**C, F, I, L**) of XCR1^{+}SIRPα^{-} cDC1 and XCR1^{-}SIRPα^{+} cDC2 among I-A/I-E^{+}CD11c^{+} leukocytes **(A-C)**, Siglec-H^{+}NK1.1^{-} pDCs and Siglec-H^{-}NK1.1^{+} NK cells among CD11c^{+}B220^{+} leukocytes **(D-F)**, CD49a^{-}CD49b^{+} NK cells and CD49a^{+}CD49b^{-} ILC1s among CD3^{-}CD19^{-}NK1.1^{+}NKp46^{+} leukocytes **(G-I)**, and RORγt^{+}CD127^{+} ILC3s among CD3^{-}CD19^{-} leukocytes **(J-L)** in Spl. Data are obtained from four to six individual samples in a single experiment. Numbers in the dot plot represent the proportion of the indicated cell populations among leukocytes. *$P < 0.05$ compared with WT mice by two-sided unpaired Student's *t*-test. (**M**) Serum productions of IL-1β, IL-2, and IL-15. Data are obtained from three individual samples in a single experiment. *P <

0.05 compared with WT mice by two-sided unpaired Student's *t*-test. All data are representative of at least three independent experiments.

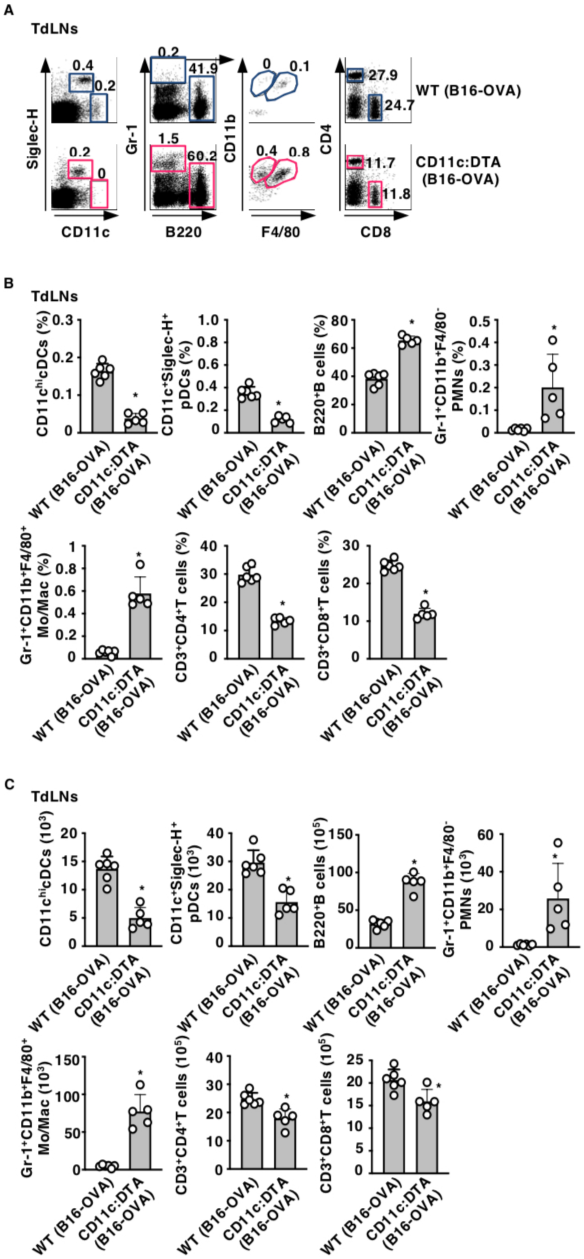

Supplementary Figure 10. Deficiency of CD11c^{hi} DCs influences the cellularity in TdLNs under tumor-bearing conditions. WT mice and CD11c:DTA mice were inoculated with B16-OVA. Cell surface expression profile **(A)**, proportion **(B)**, and absolute number **(C)** of leukocytes in TdLNs on days 18-21 after tumor inoculation. Data are obtained from five to six individual samples in a single experiment. Numbers in the dot plot represent the proportion of the indicated cell populations among leukocytes. *$P < 0.05$ compared with WT mice by two-sided unpaired Student's *t*-test. All data are representative of at least three independent experiments.

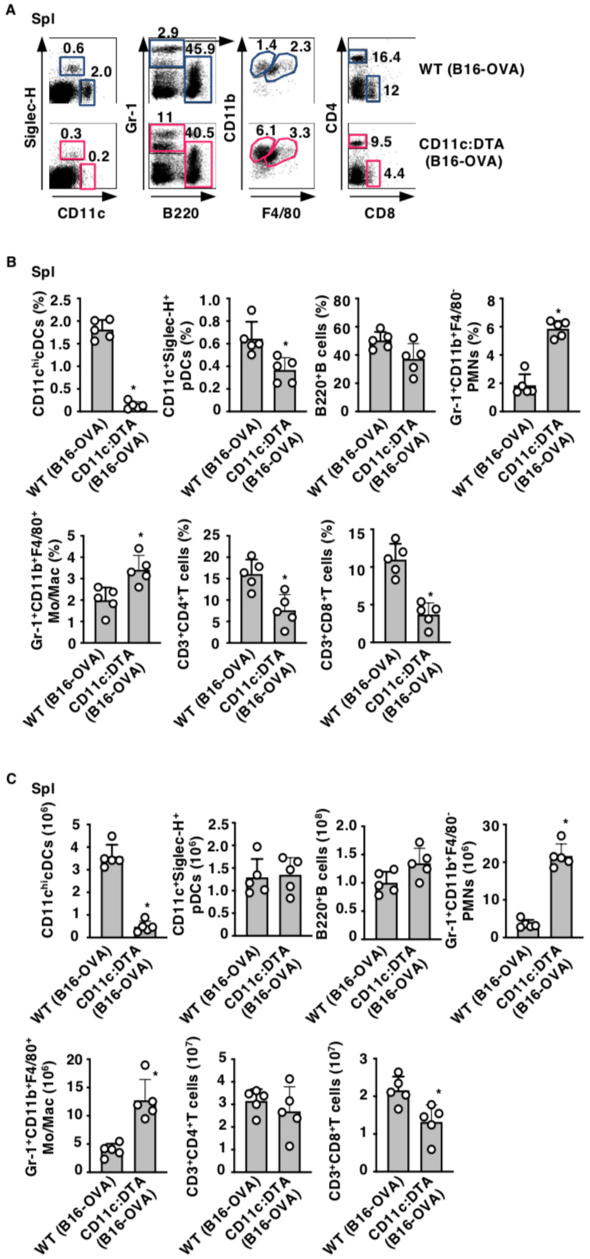

Supplementary Figure 11. Deficiency of CD11c^{hi} DCs influences the cellularity in Spl under tumor-bearing conditions. WT mice and CD11c:DTA mice were inoculated with B16-OVA. Cell surface expression profile **(A)**, proportion **(B)**, and absolute number **(C)** of leukocytes in Spl on days 18-21 after tumor inoculation. Data are obtained from five to six individual samples in a single experiment. Numbers in the dot plot represent the proportion of the indicated cell populations among leukocytes. **P* < 0.05 compared with WT mice by two-sided unpaired Student's *t*-test. All data are representative of at least three independent experiments.

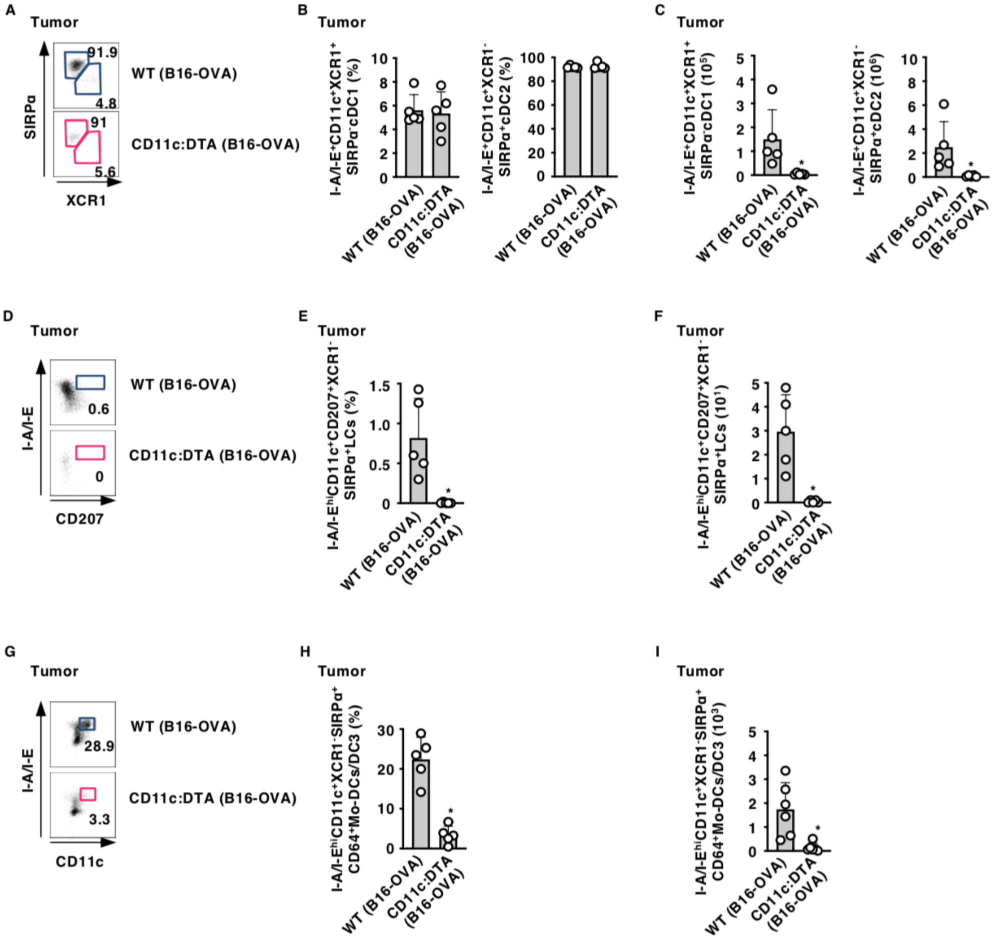

Supplementary Figure 12. Deficiency of CD11c^{hi} DCs influences the constituencies of the subsets of leukocytes in tumor tissues under tumor-bearing conditions. WT mice and CD11c:DTA mice were inoculated with B16-OVA. Cell surface expression profile (**A, D, G**), proportion (**B, E, H**), and absolute number (**C, F, I**) of XCR1^{+}SIRPα^{-} migratory cDC1 and XCR1^{-}SIRPα^{+} migratory cDC2 among I-A/I-E^{hi}CD11c^{+} leukocytes **(A-C)**, I-A/I-E^{hi}CD207^{+} LCs among CD11c^{+}XCR1^{-}SIRPα^{+} leukocytes **(D-F)**, and I-A/I-E^{hi}CD11c^{+} Mo-DCs/DC3 among XCR1^{-}SIRPα^{+}CD64^{+} leukocytes **(G-I)** in tumor tissues on days 18-21 after tumor inoculation. Data are obtained from five to six individual samples in a single experiment. Numbers in the dot plot represent the proportion of the indicated cell populations among leukocytes. *$P < 0.05$ compared with WT mice by two-sided unpaired Student's *t*-test. All data are representative of at least three independent experiments.

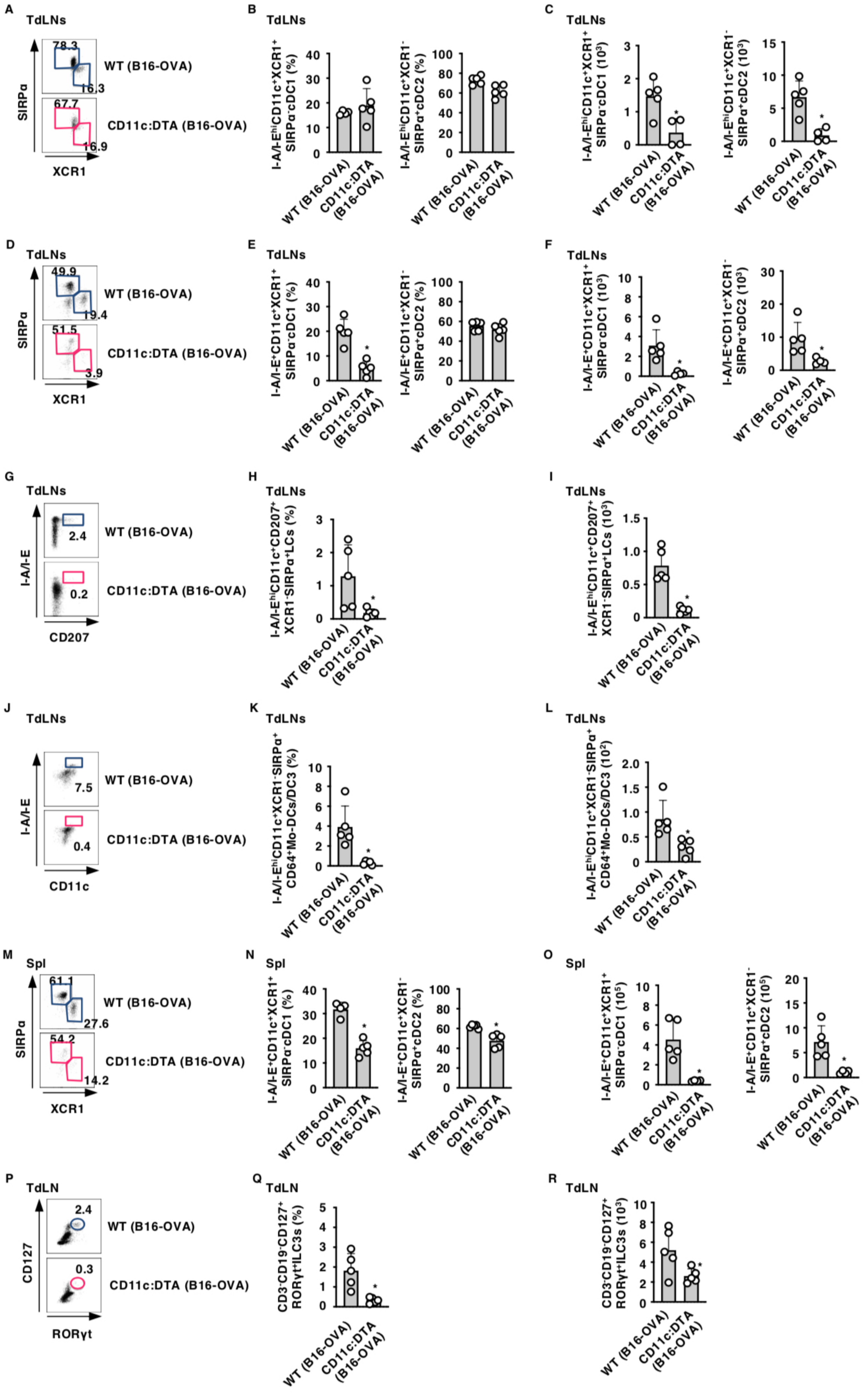
A
TdLNs
78.3
16.3
WT (B16-OVA)
67.7
16.9
CD11c:DTA (B16-OVA)
SIRPα
XCR1
B
TdLNs
I-A/I-E^hiCD11c^+XCR1^+ SIRPα^-cDC1 (%)
I-A/I-E^hiCD11c^+XCR1^- SIRPα^+cDC2 (%)
WT (B16-OVA)
CD11c:DTA (B16-OVA)
C
TdLNs
I-A/I-E^hiCD11c^+XCR1^+ SIRPα^-cDC1 (10^3)
I-A/I-E^hiCD11c^+XCR1^- SIRPα^+cDC2 (10^3)
D
TdLNs
49.9
19.4
51.5
3.9
SIRPα
XCR1
E
TdLNs
I-A/I-E^+CD11c^+XCR1^+ SIRPα^-cDC1 (%)
I-A/I-E^+CD11c^+XCR1^- SIRPα^+cDC2 (%)
F
TdLNs
I-A/I-E^+CD11c^+XCR1^+ SIRPα^-cDC1 (10^3)
I-A/I-E^+CD11c^+XCR1^- SIRPα^+cDC2 (10^3)
G
TdLNs
2.4
0.2
I-A/I-E
CD207
H
TdLNs
I-A/I-E^hiCD11c^+CD207^+ XCR1^-SIRPα^+LCs (%)
I
TdLNs
I-A/I-E^hiCD11c^+CD207^+ XCR1^-SIRPα^+LCs (10^3)
J
TdLNs
7.5
0.4
I-A/I-E
CD11c
K
TdLNs
I-A/I-E^hiCD11c^+XCR1^-SIRPα^+ CD64^+Mo-DCs/DC3 (%)
L
TdLNs
I-A/I-E^hiCD11c^+XCR1^-SIRPα^+ CD64^+Mo-DCs/DC3 (10^2)
M
Spl
61.1
27.6
54.2
14.2
SIRPα
XCR1
N
Spl
I-A/I-E^+CD11c^+XCR1^+ SIRPα^-cDC1 (%)
I-A/I-E^+CD11c^+XCR1^- SIRPα^+cDC2 (%)
O
Spl
I-A/I-E^+CD11c^+XCR1^+ SIRPα^-cDC1 (10^5)
I-A/I-E^+CD11c^+XCR1^- SIRPα^+cDC2 (10^5)
P
TdLN
2.4
0.3
CD127
RORγt
Q
TdLN
CD3^-CD19^-CD127^+ RORγt^+ILC3s (%)
R
TdLN
CD3^-CD19^-CD127^+ RORγt^+ILC3s (10^3)

Supplementary Figure 13. Deficiency of $CD11c^{hi}$ DCs influences the constituencies of the subsets of leukocytes in TdLNs and Spl under tumor-bearing conditions. WT mice and CD11c:DTA mice were inoculated with B16-OVA. **(A-L)** Cell surface expression profile (**A, D, G, J**), proportion (**B, E, H, K**), and absolute number (**C, F, I, L**) of $XCR1^+SIRP\alpha^-$ migratory cDC1 and $XCR1^-SIRP\alpha^+$ migratory cDC2 among $I\text{-}A/I\text{-}E^{hi}CD11c^+$ leukocytes **(A-C)**, $XCR1^+SIRP\alpha^-$ resident cDC1 and $XCR1^-SIRP\alpha^+$ resident cDC2 among $I\text{-}A/I\text{-}E^+CD11c^+$ leukocytes **(D-F)**, $I\text{-}A/I\text{-}E^{hi}CD207^+$ LCs among $CD11c^+XCR1^-SIRP\alpha^+$ leukocytes (**G-I**), and $I\text{-}A/I\text{-}E^{hi}CD11c^+$ Mo-DCs/DC3 among $XCR1^-SIRP\alpha^+CD64^+$ leukocytes (**J-L**) in TdLNs on days 18-21 after tumor inoculation. Data are obtained from four to five individual samples in a single experiment. Numbers in the dot plot represent the proportion of the indicated cell populations among leukocytes. $*P < 0.05$ compared with WT mice by two-sided unpaired Student's *t*-test. **(M-O)** Cell surface expression profile (**M**), proportion (**N**), and absolute number (**O**) of $XCR1^+SIRP\alpha^-$ cDC1 and $XCR1^-SIRP\alpha^+$ cDC2 among $I\text{-}A/I\text{-}E^+CD11c^+$ leukocytes in Spl on days 18-21after tumor inoculation. Data are obtained from five to six individual samples in a single experiment. Numbers in the dot plot represent the proportion of the indicated cell populations among leukocytes. $*P < 0.05$ compared with WT mice by two-sided unpaired Student's *t*-test. **(P-R)** Cell surface expression profile (**P**), proportion (**Q**), and absolute number (**R**) of $ROR\gamma t^+CD127^+$ ILC3s among $CD3^-CD19^-$ leukocytes in TdLNs on days 18-21 after tumor inoculation. Data are obtained from five to six individual samples in a single experiment. Numbers in the dot plot represent the proportion of the indicated cell populations among leukocytes. $*P < 0.05$ compared with each group of WT mice by two-sided unpaired Student's *t*-test. All data are representative of at least three independent experiments.

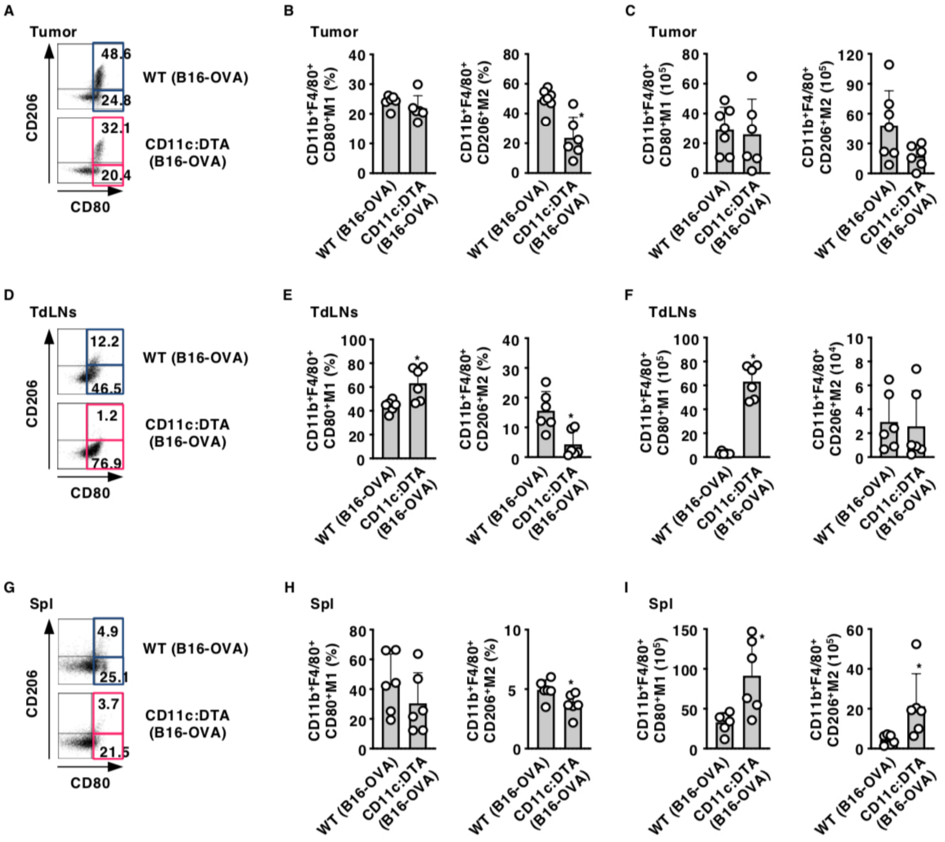

Supplementary Figure 14. Deficiency of CD11c^{hi} DCs influences the constituencies of the subsets of macrophages in under tumor-bearing conditions. WT mice and CD11c:DTA mice were inoculated with B16-OVA. Cell surface expression profile **(A, D, G)**, proportion **(B, E, H)**, and absolute number **(C, F, I)** of the subsets of macrophages among CD11b$^+$F4/80$^+$ leukocytes in tumor tissues **(A-C)**, TdLNs **(D-F)**, and Spl **(G-I)** on days 18-21 after tumor inoculation. Data are obtained from five to seven individual samples in a single experiment. Numbers in the dot plot represent the proportion of the indicated cell populations among leukocytes. *$P < 0.05$ compared with WT mice by two-sided unpaired Student's *t*-test. All data are representative of at least three independent experiments.

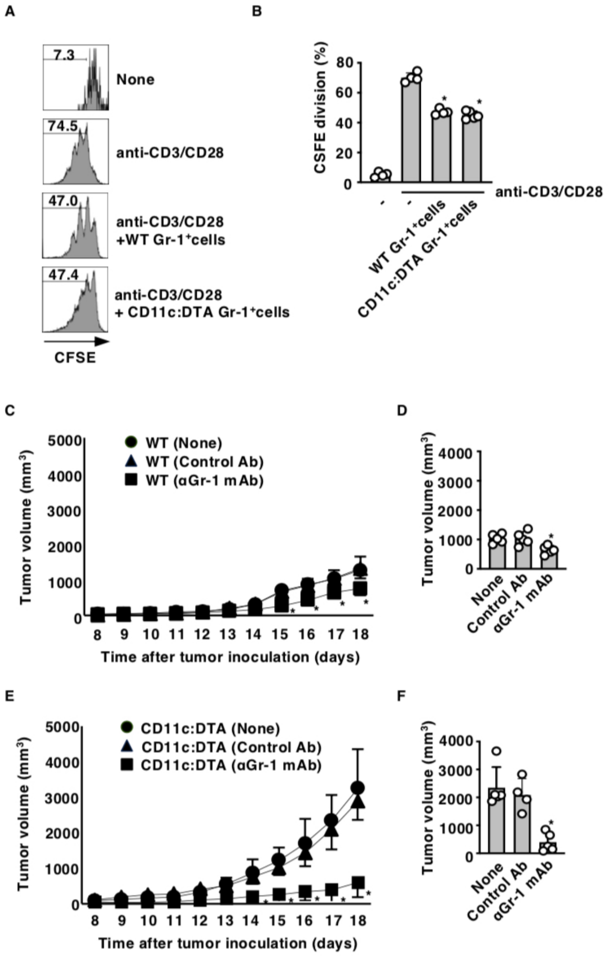

Supplementary Figure 15. Role of MDSCs in the progression of tumor under the deficiency of CD11c^{hi} DCs. **(A, B)** Cell dividing profile **(A)** and proportion **(B)** of eFluor™ 670-labelled CD4^{+} T cells stimulated with or without anti-CD3/CD28 mAbs in the presence or absence of Gr-1^{+} cells in Spl obtained from CD11c:DTA mice that had been inoculated with B16-OVA for 4 days. Data are obtained from four to six individual samples in a single experiment. Numbers in the histogram represent the proportion of the dividing cells. *$P < 0.05$ compared with CD4^{+} T cells stimulated with anti-CD3/CD28 mAbs in the absence of Gr-1^{+} cells by two-sided unpaired Student's *t*-test. (**C-F**) WT mice (**C, D**) and CD11c:DTA mice (**E, F**) that had inoculated with B16-OVA were treated with or without anti-Gr-1 mAb or control Ab, and tumor growth was monitored. **(C, E)** Tumor volume in WT mice (**C**) and CD11c:DTA mice (**E**) for 18 days. **(D, F)** Tumor volume in WT mice (**D**) and CD11c:DTA mice (**F**) on 17 days; n = 5 per group. *$P < 0.05$ compared with each group of untreated mice by two-sided unpaired Student's *t*-test. All data are representative of at least three independent experiments.

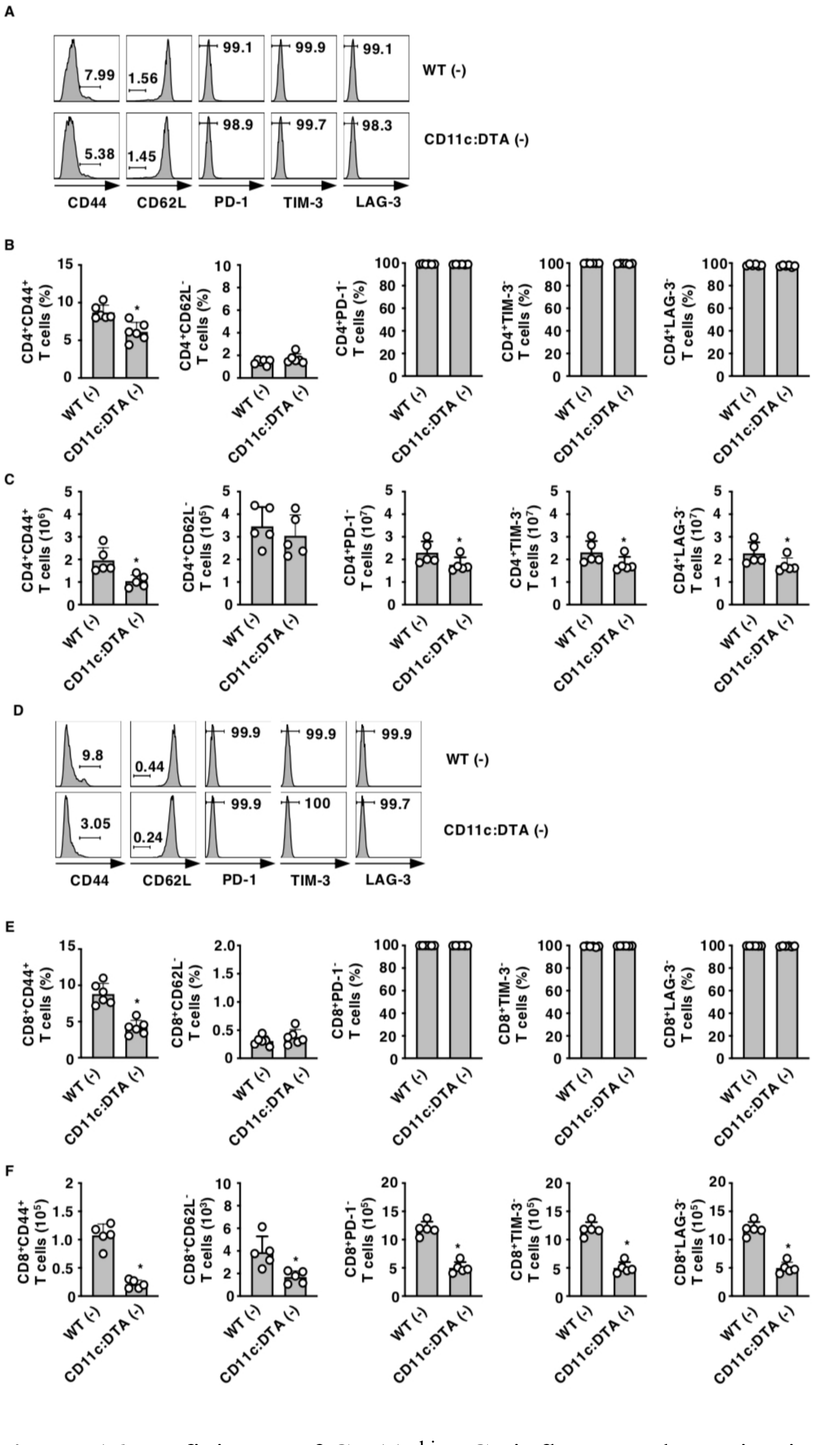

Supplementary Figure 16. Deficiency of CD11c^{hi} DCs influences the activation status of T cells in LNs under homeostatic conditions. Cell surface expression profile **(A, D)**, proportion **(B, E)**, and absolute number **(C, F)** of CD4^{+} T cells **(A-C)** and CD8^{+} T cells **(D-F)** in LNs. Data are obtained from five to six individual samples in a single experiment. Numbers in the histogram represent the proportion of the indicated cell populations. *$P < 0.05$ compared with WT mice by two-sided unpaired Student's *t*-test. All data are representative of at least three independent experiments.

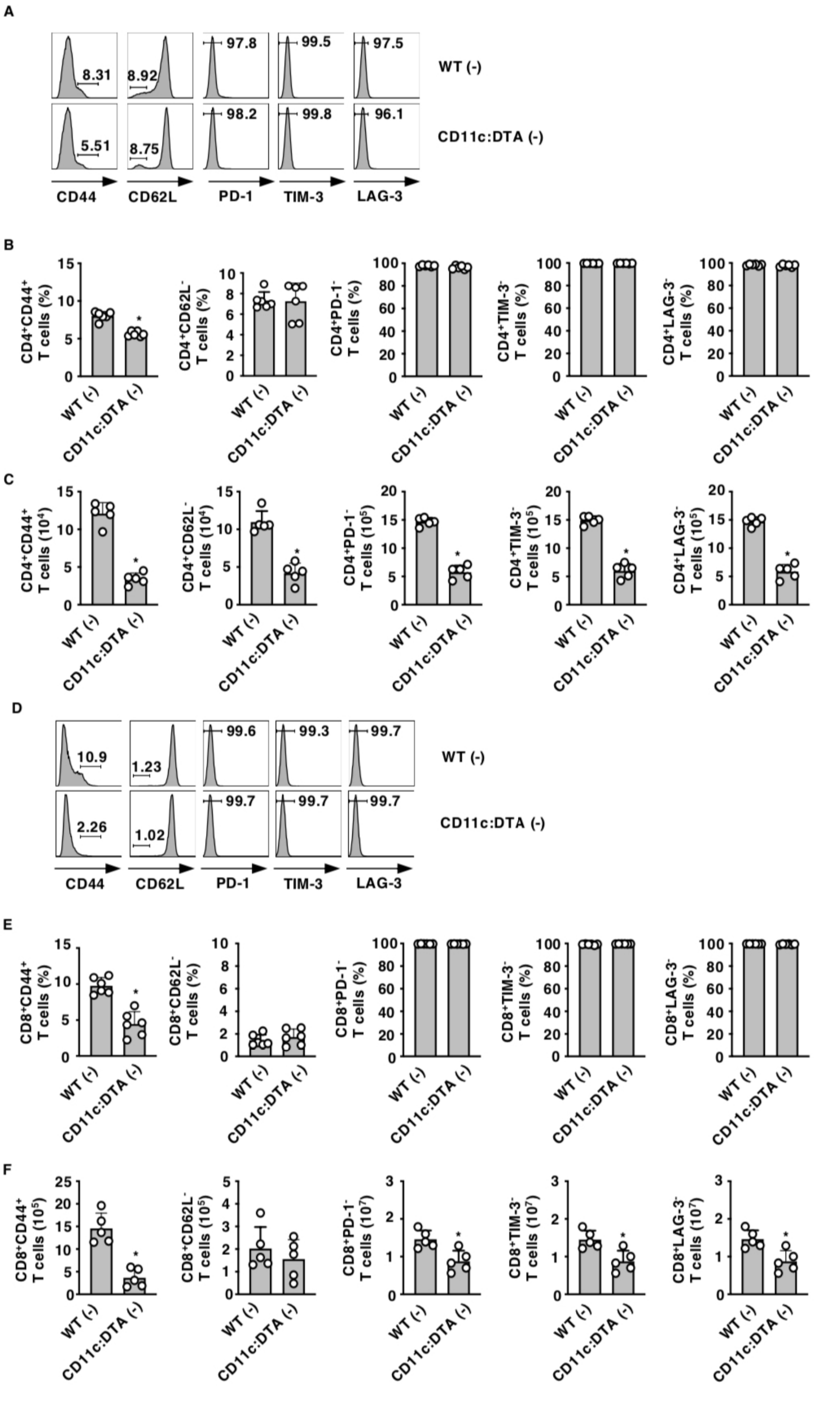

Supplementary Figure 17. Deficiency of CD11c^{hi} DCs influences the activation status of T cells in Spl under homeostatic conditions. Cell surface expression **(A, D)**, proportion **(B, E)**, and absolute number **(C, F)** of CD4^{+} T cells **(A-C)** and CD8^{+} T cells **(D-F)** in Spl. Data are obtained from five to six individual samples in a single experiment. Numbers in the histogram represent the proportion of the indicated cell populations. *P < 0.05 compared with WT mice by two-sided unpaired Student's *t*-test. All data are representative of at least three independent experiments.

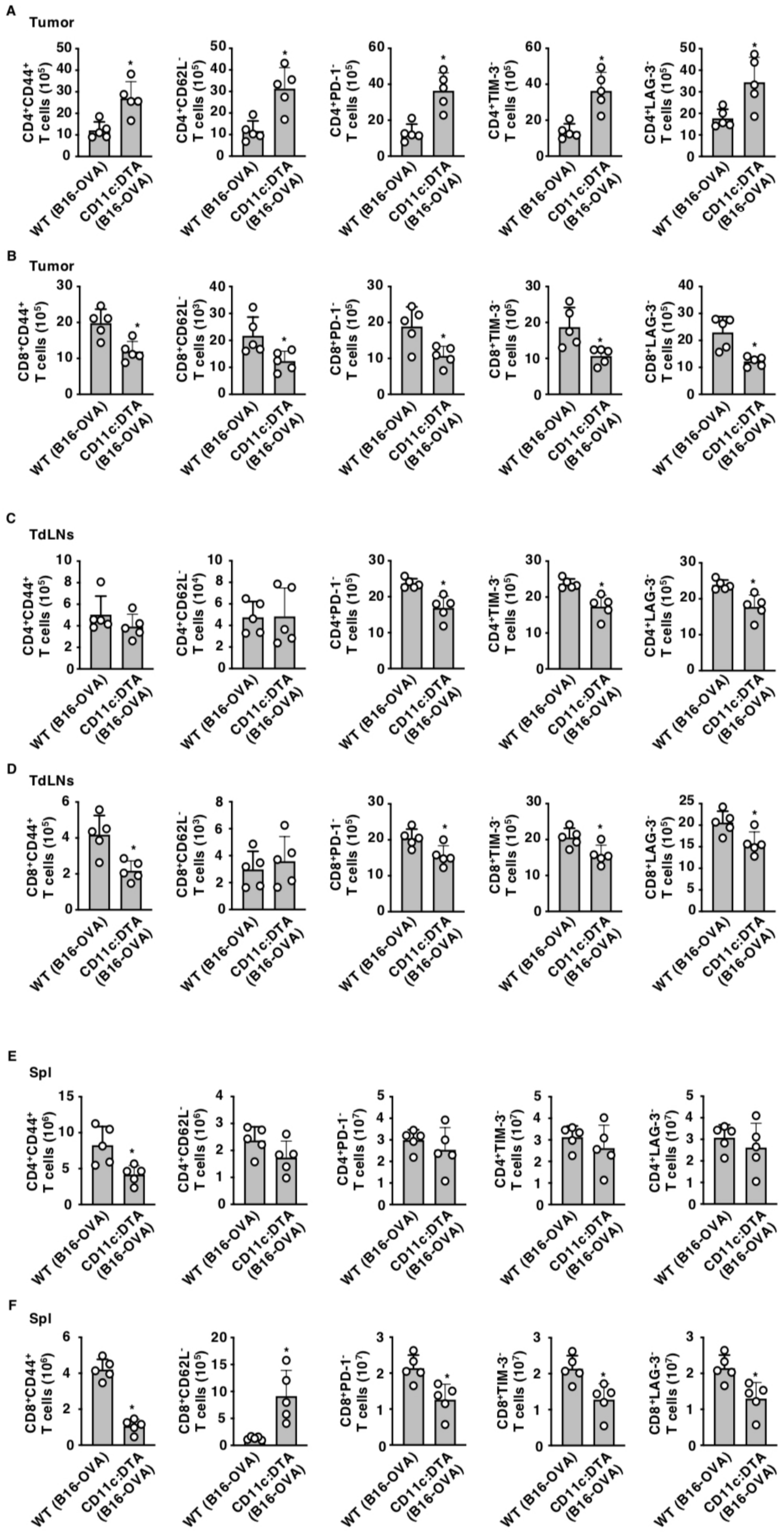

A
Tumor
B
Tumor
C
TdLNs
D
TdLNs
E
Spl
F
Spl
WT (B16-OVA)
CD11c:DTA (B16-OVA)

Supplementary Figure 18. Deficiency of CD11c^{hi} DCs controls the activation status of T cells under tumor-bearing conditions. WT mice and CD11c:DTA mice were inoculated with B16-OVA. Absolute number of CD4^{+} T cells **(A, C, E)** and CD8^{+} T cells **(B, D, F)** in tumor tissues **(A, B)**, TdLNs **(C, D)**, and Spl **(E, F)** on days 18-21 after tumor inoculation. Data are obtained from five to eight individual samples in a single experiment. Numbers in the histogram represent the proportion of the indicated cell populations. *$P < 0.05$ compared with WT mice by two-sided unpaired Student's *t*-test. All data are representative of at least three independent experiments.

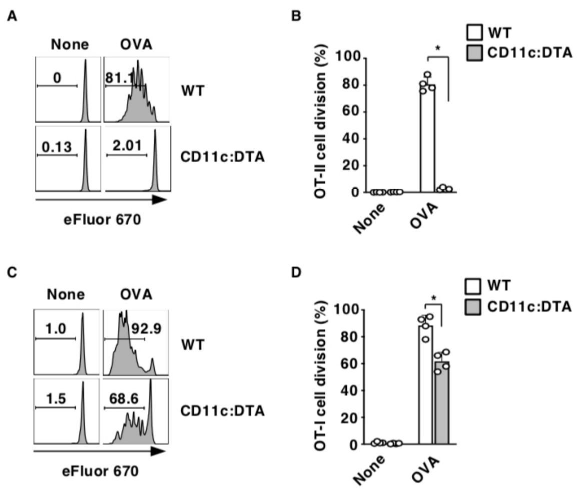

Supplementary Figure 19. Deficiency of CD11c^{hi} DCs reduces the response of Ag-specific T cells in LNs under homeostatic conditions. WT mice and CD11c:DTA mice were adoptively transferred with eFluor™ 670-labelled CD45.1^{+}OT-II CD4^{+} T cells **(A, B)** or CD45.1^{+}OT-I CD8^{+} T cells **(C, D)**, and then mice were systemically administered OVA protein. Cell dividing profile **(A, C)** and proportion **(B, D)** at 2 days after the administration. Data are obtained from four to six individual samples in a single experiment. Numbers in the histogram represent the proportion of the dividing cells. *$P < 0.01$ (**B**) or *$P < 0.05$ (**D**) compared with each group of WT mice by two-sided unpaired Student's *t*-test. All data are representative of at least three independent experiments.

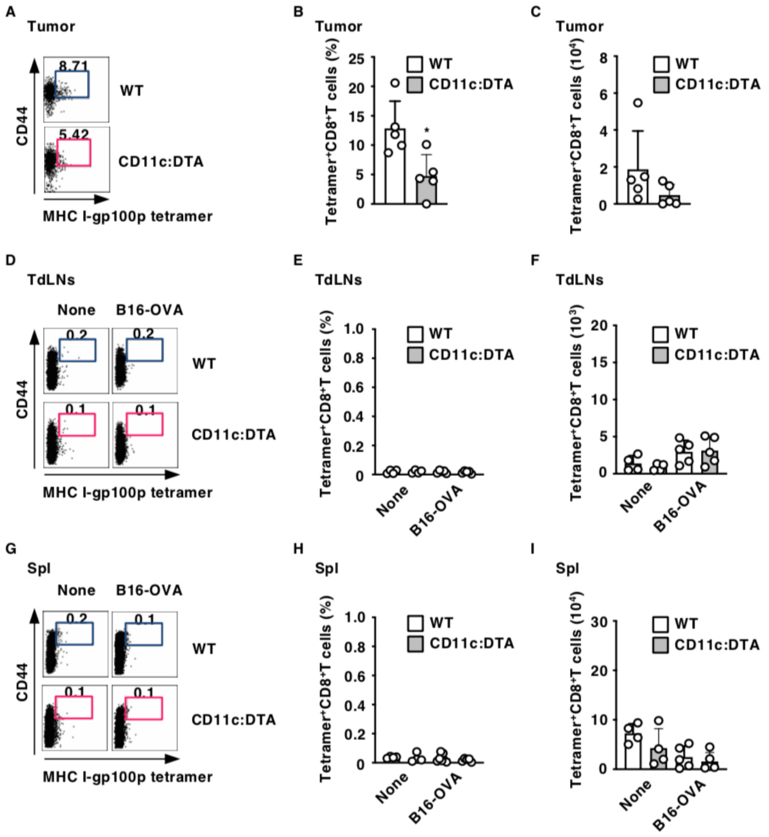

Supplementary Figure 20. Deficiency of CD11c^{hi} DCs decreases the generation of tumor-specific CTLs under tumor-bearing conditions. WT mice and CD11c:DTA mice were inoculated with or without B16-OVA. Cell surface expression profile **(A, D, G),** proportion **(B, E, H)**, and absolute number **(C, F, I)** of MHC I-gp100 tetramer^{+}CD44^{high}CD8^{+} T cells among CD8^{+} T cells in tumor tissues **(A-C)**, TdLNs **(D-F)**, and Spl **(G-I)** on days 18-21 after tumor inoculation. Data are obtained from four to six individual samples in a single experiment. Numbers in the dot plot represent the proportion of the indicated cell populations. *$P < 0.05$ compared with WT mice by two-sided unpaired Student's *t*-test. All data are representative of at least three independent experiments.

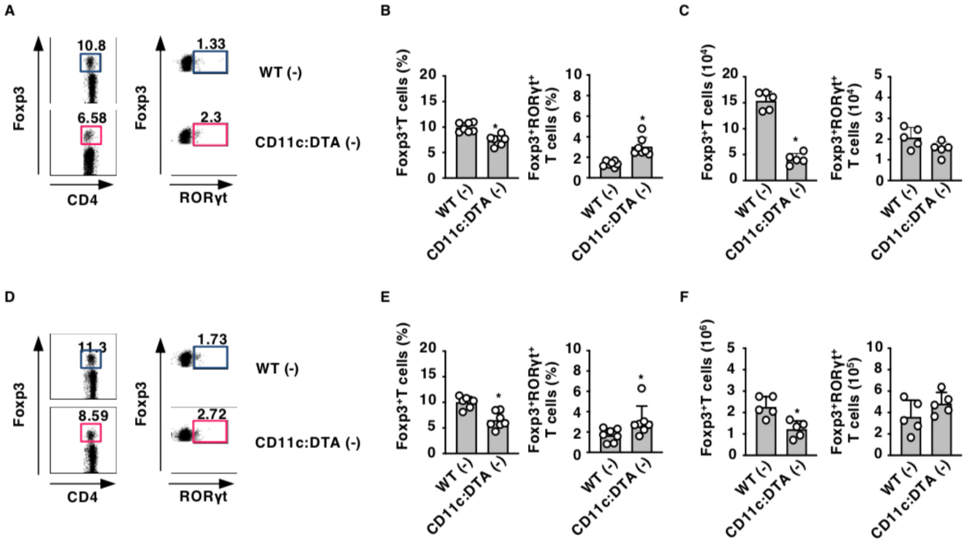

Supplementary Figure 21. Deficiency of CD11c^{hi} DCs influences the proportion of CD4^{+}Foxp3^{+} T$_{reg}$ cells under homeostatic conditions. Cell surface expression profile **(A, D)**, proportion **(B, E)**, and absolute number **(C, F**) in LNs **(A-C)**, and Spl **(D-F)**. Data are obtained from five to seven individual samples in a single experiment. Numbers in the dot plot represent the proportion of the indicated cell populations. *P < 0.05 compared with WT mice by two-sided unpaired Student's *t*-test. All data are representative of at least three independent experiments.
